# Whole‐genome sequencing and antigenic analysis of the first equine influenza virus identified in Turkey

**DOI:** 10.1111/irv.12485

**Published:** 2018-02-08

**Authors:** Jacinta Gahan, Marie Garvey, Sarah Gildea, Emre Gür, Anil Kagankaya, Ann Cullinane

**Affiliations:** ^1^ Virology Unit Irish Equine Centre Naas Ireland; ^2^ Head of Equine Health and Veterinary Services Department Jockey Club of Turkey Istanbul Turkey; ^3^ Department of Surgery Ankara University Faculty of Veterinary Medicine Ankara Turkey

**Keywords:** American lineage, equine influenza, Florida clade 2, genome sequencing, Turkey

## Abstract

**Background:**

In 2013, there was an outbreak of acute respiratory disease in racehorses in Turkey. The clinical signs were consistent with equine influenza (EI).

**Objective:**

The aim was to confirm the cause of the outbreak and characterise the causal virus.

**Methods:**

A pan‐reactive influenza type A real‐time RT‐PCR and a rapid antigen detection kit were used for confirmatory diagnosis of equine influenza virus (EIV). Immunological susceptibility to EIV was examined using single radial haemolysis and ELISA. Antigenic characterisation was completed by haemagglutinin inhibition using a panel of specific ferret antisera. Genetic characterisation was achieved by whole‐genome sequencing using segment‐specific primers with M13 tags.

**Results:**

A H3N8 EIV of the Florida clade 2 sublineage (FC2) was confirmed as the causal agent. The index cases were unvaccinated and immunologically susceptible. Phylogenetic analysis of the HA1 and NA genes demonstrated that A/equine/Ankara/1/2013 clustered with the FC2 strains circulating in Europe. Antigenic characterisation confirmed the FC2 classification and demonstrated the absence of significant drift. Whole‐genome sequencing indicated that A/equine/Ankara/1/2013 is most closely related to the viruses described as the 179 group based on the substitution I179V in HA1, for example A/equine/East Renfrewshire/2/2011, A/equine/Cambremer/1/2012 and A/equine/Saone et Loire/1/2015. The greatest diversity was observed in the NS1 segment and the polymerase complex.

**Conclusions:**

The first recorded outbreak of EI in Turkey was caused by an FC2 virus closely related to viruses circulating in Europe. Antigenic and genetic characterisation gave no indication that the current OIE recommendations for EI vaccine composition require modification.

## 1 INTRODUCTION

Equine influenza virus (EIV) of the H3N8 subtype is associated with an acute respiratory disease which has immense economic relevance due to its highly contagious nature and potential to disrupt equestrian events.^1^, ^2^ Each equine influenza (EI) virion contains 8 segments of negative sense viral RNA named; PB2, PB1, PA, HA, NP, NA, M and NS. These segments encode at least 10 proteins which include the surface glycoproteins; haemagglutinin (HA) and neuraminidase (NA), the matrix ion channel (M2), the matrix protein (M1), three polymerases (PA, PB1 and PB2), the structural nucleoprotein (NP), the non‐structural protein (NS1), the nuclear export protein (NEP), an accessory pro‐apoptotic protein (PB1‐F2)^3^ and a recently discovered PA‐X protein.^4^ The HA glycoprotein is particularly important in the evolution of EIV as it stimulates a strong humoral antibody response following infection.^5^, ^6^ Mutations in the HA lead to antigenic drift and emergence of antigenically distinct EIV lineages. Subsequent to EI epizootics in 1989, phylogenetic analysis of the HA gene revealed divergence of the circulating H3N8 viruses into the Eurasian and American lineages.^7^ In the 1990s, the American lineage diverged into the South America, Kentucky and Florida sublineages.^8^ More recently, the Florida sublineage has predominated and evolved into 2 additional distinct clades, Florida clades 1 and 2 (FC1, FC2).^5^ FC1 and FC2 viruses are prevalent in the USA and Europe, respectively.^1^ However, both have caused outbreaks elsewhere FC1 in South Africa,^9^ Japan,^10^ Australia^11^ and Europe^5^, ^12^, ^13^ and FC2 in China^14^ Mongolia^15^ and India.^16^

In endemic populations, EIV persists due to the existence of susceptible partially vaccinated or unvaccinated horses and compromised vaccine effectiveness, which occurs as a result of antigenic mismatch between vaccine and field strain viruses.^17^, ^18^ Experimentally infected animals vaccinated with a heterologous vaccine strain shed virus for longer than those that received a homologous vaccine.^19^ In countries which are free from EI, the virus has frequently been introduced by the importation of infected vaccinated horses.^20^, ^21^ Thus, it is extremely important that the equine industry has access to epidemiologically relevant vaccines. To achieve this aim, phylogenetic and antigenic analyses of viruses circulating worldwide are undertaken annually by the OIE (World Organisation for Animal Health) Expert Surveillance Panel (ESP). Since 2010, the OIE has recommended the inclusion of FC1 represented by A/equine/South Africa/04/2003‐like or A/eq/Ohio/2003‐like viruses and FC2 represented by A/equine/Richmond/1/2007‐like viruses in EI vaccines. The aim of this study was to confirm the cause of an outbreak of influenza‐like respiratory disease in horses stabled at a racetrack in Ankara, Turkey, and to characterise the causal virus.

## 2 MATERIALS AND METHODS

### 2.1 Clinical detection

Nasopharyngeal swabs and serum samples were collected from 7 clinically affected horses. The nasopharyngeal swabs were tested by pan‐reactive influenza type A real‐time RT‐PCR (qRT‐PCR) described by Heine et al 2007^22^ and the Directigen EZ FluA + B^™^ rapid detection kit (BD Diagnostics, Oxford, England). Serum samples were tested by single radial haemolysis (SRH) 23 and the IDScreen influenza A antibody ELISA (IDvet, Grabels, France), a multispecies competition ELISA that detects antibodies against the internal nucleocapsid of the influenza A virus.

### 2.2 Virus isolation

Isolation of viruses from three EI qRT‐PCR‐positive nasopharyngeal swabs (A/equine/Ankara/1/2013, A/equine/Ankara/2/2013 and A/equine/Ankara/3/2013) was carried out in 10‐day‐old embryonated hens’ eggs.^24^ A/equine/Ankara/1/2013 had the highest HA titre following initial passage and was therefore selected for repeat passage to reach the required concentration for sequencing (HA of 1:128).

### 2.3 Antigenic characterisation

A/equine/Ankara/1/2013 was antigenically characterised by the haemagglutination inhibition (HI) test using clade‐specific ferret antisera pre‐treated with heat and periodate.^5^ In summary, 4 HA units of each virus was tested with serial dilutions of ferret antisera raised against A/equine/Newmarket/1/93 and A/equine/Newmarket/2/93 (representative strains of American and Eurasian lineages—obtained from the OIE Reference Laboratory, Animal Health Trust, Newmarket, UK), A/equine/South‐Africa/4/03 and A/equine/Donegal/09 (representative strains of the FC1 sublineage), and A/equine/Meath/07 and A/equine/Kildare/12 (representative strains of the FC2 sublineage). Geometric mean titres were calculated for three HI tests for each combination.

### 2.4 Whole‐genome sequencing

A/equine/Ankara/1/2013 was passaged twice in embryonated hens’ eggs and diluted to 10^6^ EID_50_/mL. RNA was extracted using QIAamp Viral RNA Mini Kit (Qiagen, Hilden, Germany). For whole‐genome sequencing (WGS), one‐step PCR was undertaken using SuperScript^™^ III One‐Step RT‐PCR System with Platinum^®^ Taq High Fidelity (Invitrogen, Carlsbad, California, USA) using overlapping M‐13 labelled primers for each of the 8 EIV segments.^25^ An additional NA primer (5′‐GCCTCACAAAGTGGTTC‐3′) was designed to obtain the start of the NA gene.

Thermocycling conditions were as follows: reverse transcription at 55°C (30 minutes) and initial denaturation at 94°C (2 minutes), 40 cycles of denaturation at 94°C (60 seconds), primer annealing at 45‐60°C (10 seconds), elongation at 60°C (60 seconds) and final elongation at 60°C for 5 minutes. PCR products were visualised on a 1% agarose gel stained with Sybersafe (Invitrogen). PCR amplicons were purified using a QIAquick PCR purification kit (Qiagen) and sequenced using Sanger dideoxynucleotide sequencing technology (GATC‐Biotech, Cologne, Germany).

Genome segments were assembled using Seqman version 14.1.0 (118) 412, DNASTAR, Madison, WI. Multiple nucleotide, and amino acid sequence alignments of each segment were constructed using the ClustalW^26^ accessory application in BioEdit sequence alignment editor version 7.2.5.^27^ Maximum‐likelihood phylogenetic trees were constructed using MEGA7 version 7.0.14^28^ with HA1 and NA gene sequences mined from the NCBI GenBank and GISAID databases 29 (see Table S1 for accession codes). The optimum model for each tree was chosen based on the lowest Bayesian information criterion scores. Following phylogenetic classification based on the HA1 gene, the genome sequence of A/equine/Ankara/1/2013 was aligned with the OIE recommended FC2 representative vaccine strain A/equine/Richmond/1/2007. Amino acid changes observed for A/equine/Ankara/1/2013 were then compared to other European strains circulating in 2009‐2015 to track evolution of EIV (see Table S2 for accession codes).

## 3 RESULTS

### 3.1 Disease outbreak

Between 11 July 2013 and 07 August 2013, 76 of 1143 (6.6%) horses stabled at the racetrack in Ankara were clinically diagnosed by veterinarians as being acutely affected with EIV. Although racehorses in Ankara had been routinely vaccinated with Equilis Prequenza (MSD Animal Health) at six monthly intervals, the majority of clinically affected horses in this instance had not been vaccinated as they were yet to race. The official notification of EIV outbreak was made to the OIE in July 2013, and disease control measures were implemented; affected horses were quarantined, and temporary movement restrictions were imposed.

### 3.2 Laboratory confirmation

Equine influenza virus was detected by qRT‐PCR in 7 nasopharyngeal swabs obtained from affected horses. All the positive field samples gave low C_*t*_ values (23.4‐29.3). The rapid horse‐side detection kit Directigen EZ Flu A + B ^™^ detected EIV in 6 of the 7 nasopharyngeal swabs. Of the 7 qRT‐PCR‐positive samples, the Directigen EZ Flu A + B ^™^‐negative swab had the highest C_*t*_ value (C_*t*_ = 29.3). All 7 horses tested seronegative by SRH (no haemolysis) and the IDScreen influenza A antibody ELISA (competition % ≥50%) indicating their immunological naïve status and susceptibility to EIV.

### 3.3 Antigenic characterisation

Antigenic characterisation identified A/equine/Ankara/1/2013 as a FC2 strain of EIV (Table 1). A panel of specific antisera raised against FC2 viruses demonstrated similarly high HI titres for A/equine/Ankara/1/2013 as they did against their homologous virus. Additionally, up to an eightfold higher titre was observed with A/equine/Ankara/1/2013 for FC2 than FC1 antisera which confirmed the FC2 classification.

**Table 1:**
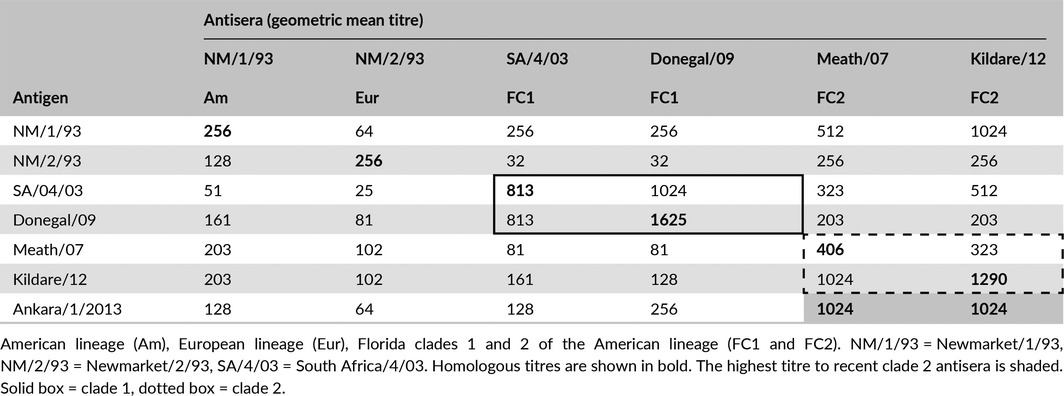
Antigenic characterisation of A/equine/Ankara/1/2013 using ferret antisera

### 3.4 Phylogenetic classification (HA1 and NA)

**Figure 1:**
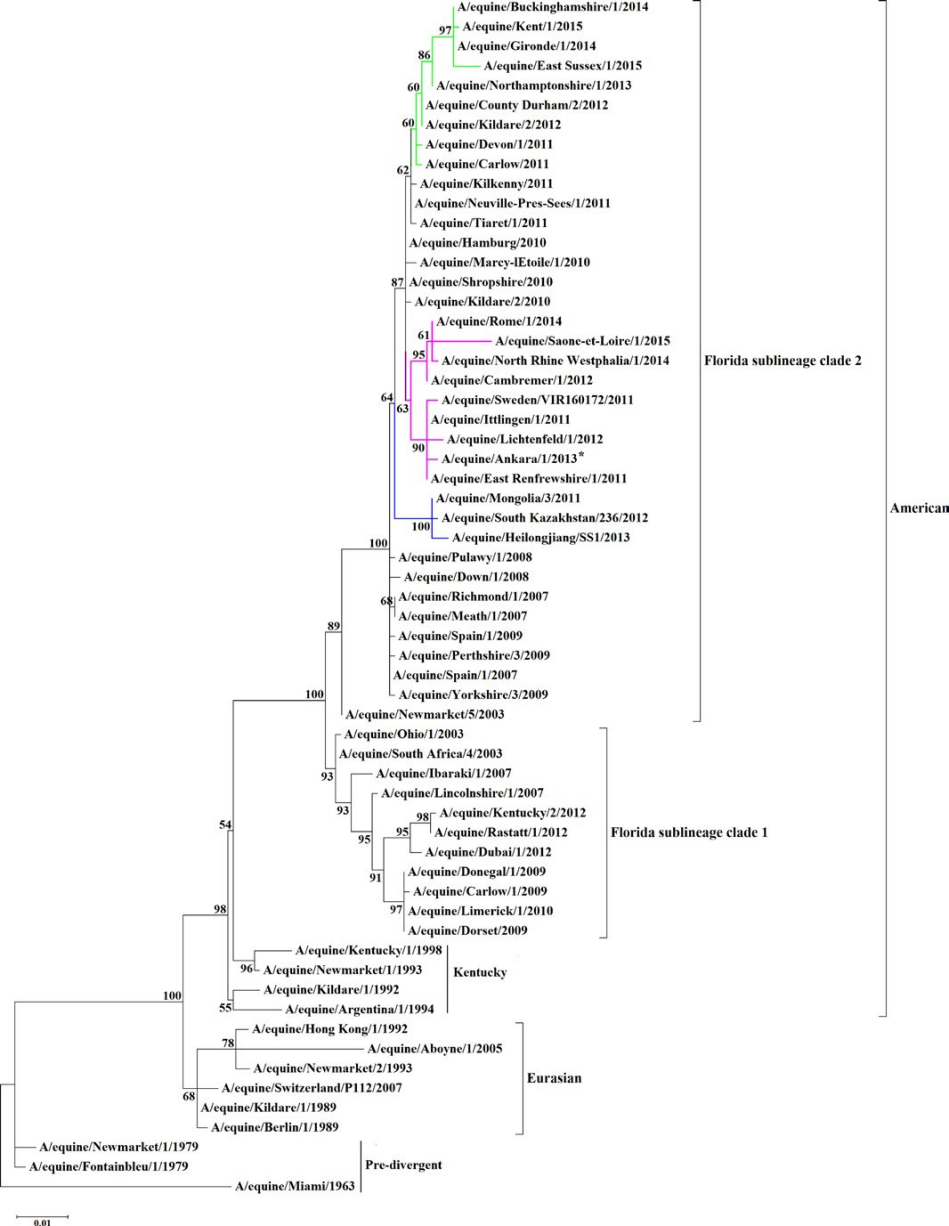
Maximum‐likelihood tree of HA1 nucleotide sequences. Phylogenetic tree of HA1 nucleotide sequences encoded by EIV subtype H3N8. Bootstrap values obtained after 1000 replicates are shown at major nodes. Florida clade 2 groups, 144 (green), 179 (pink) and Asian (blue) are highlighted. Accession numbers for the sequences are listed in Table S1

**Figure 2:**
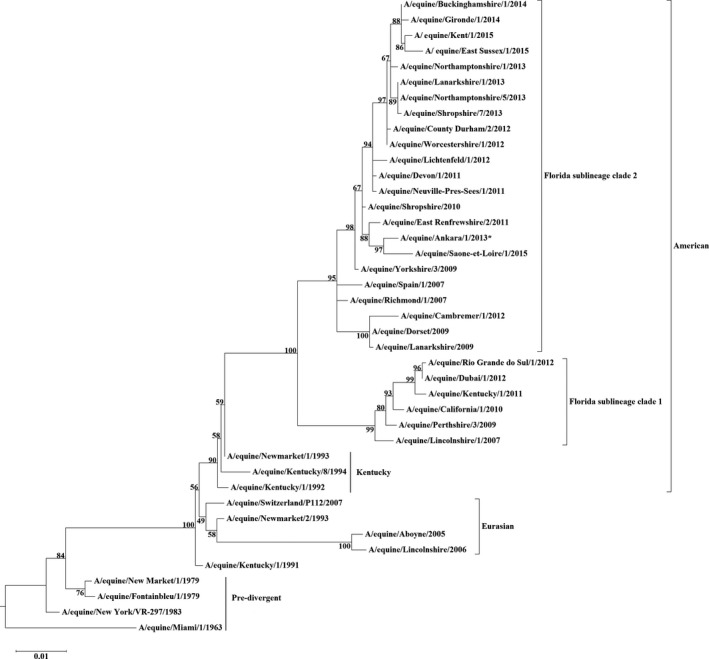
Maximum‐likelihood tree of NA nucleotide sequences. Phylogenetic analysis of NA nucleotide sequences encoded by EIV subtype H3N8. Bootstrap values obtained after 1000 replicates are shown at major nodes. Accession numbers for the sequences are listed in Table S1

A maximum‐likelihood phylogenetic tree confirmed that A/equine Ankara/1/2013 is an FC2 strain as it clustered with other isolates in that clade that have the I179V substitution, that is the “179 group” (Figure 1). Similarly, the phylogenetic analysis of the nucleotide sequence encoding NA indicated that A/equine/Ankara/1/2013 was most similar to the UK isolate A/equine/East Renfrewshire/2/2011 and the French isolate A/equine/Saone et Loire/1/2015 (Figure 2).

### 3.5 Whole‐genome sequencing

Whole‐genome sequencing was achieved using overlapping M‐13 labelled segment‐specific primer pairs. The data generated have been deposited in GenBank with accession numbers MF067524‐MF067531. Firstly, the predicted amino acid sequences of the assembled genome segments were aligned with A/equine/Richmond/1/2007 to ascertain amino acid changes from the OIE recommended FC2 vaccine strain (Figs S3.1‐S3.12, Table 2). Within the RNA‐dependent polymerase complex (PB2, PB1 and PA), a total of 3 (S107N, R389K and R586K), 3 (E75K, T221A and H456Y) and 7 (K22R, D27N, A100T, K158R, M210T, D394N and R531K) amino acid changes were observed, respectively. The A/equine/Ankara/1/2013 PA‐X gene is full length unlike A/equine/Richmond/1/2007 which is truncated, but no additional amino acid changes were observed. The surface glycoproteins HA and NA have 5 and 6 amino acid changes, respectively (HA: P103L, V112I, I179V, I214T, E291D, NA: G47R, R109K, T381I, I410V, K415R, T434S) (Table 2). The non‐structural proteins PB1‐F2 and NS1 have 2 (D50V and R75H) and 5 (H17N, E26G, T80I, V111A and Y207H) amino acid changes, respectively. The structural NP, M1 and NEP proteins have 2 (M239V and N473S), 1 (T168I)) and 1 (R86G) amino acid changes, respectively. The M2 ion channel protein has 2 amino acid changes (D21G and V68I).

**Table 2:**
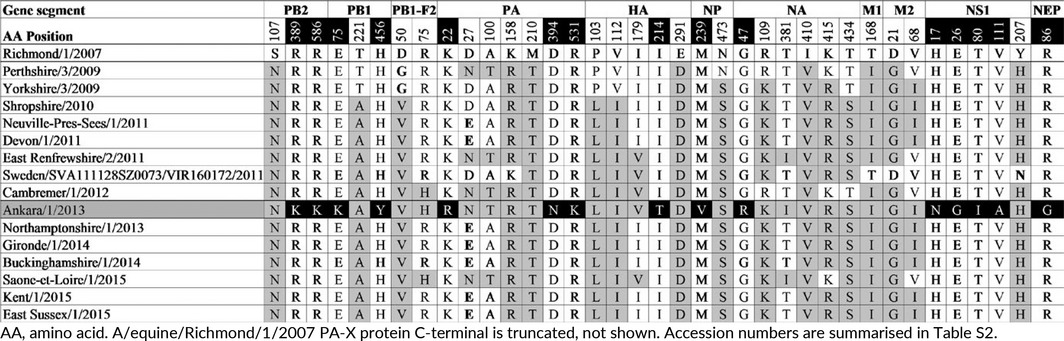
Amino acid differences between A/equine/1/Richmond/2007 and A/equine/Ankara/1/2013 and comparison to recent European Florida clade 2 isolates (2009‐2015)

To monitor the evolutionary divergence of FC2 viruses from the recommended vaccine strain, the substitutions between A/equine/Ankara/1/2013 and A/equine/Richmond/1/2007 were then compared to other FC2 strains circulating in Europe from 2009 to 2015. The results are summarised in Table 2. The majority of the substitutions observed in the surface glycoproteins are conserved in European FC2 viruses (Figs S1 and S2). Comparison of HA amino acid sequences demonstrated that A/equine/Ankara/1/2013 HA has the fixed substitutions that emerged around 2007 (E291D) and 2010 (P103L and V112I).^5^, ^30^ In 2011, two subpopulations were identified with an additional change at either position 144 or at 179.^30^ A/equine/Ankara/1/2013 has the substitution I179V similar to A/equine/East Renfrewshire/2/2011, A/equine/Sweden/SVA111128SZ0073/VIR160172/2011, A/equine/Cambremer/1/2012 and A/equine/Saone et Loire/1/2015. A/equine/Ankara/1/2013 also has a substitution I214T which appears to be unique. Four of the NA amino acid substitutions R109K, T381I, I410V, K415R and T434S are conserved in the majority of European FC2 viruses since 2009/2010. The T381I substitution is less common but present in A/equine/East Renfrewshire/2/2011 and A/equine/Saone et Loire/1/2015.

One of the substitutions in the NP appears fixed since 2009, but the other appears to be unique as does the single substitution in NEP. The single substitution in M1 is highly conserved as are the two substitutions in M2.

In contrast to the glycoproteins and other structural proteins, the majority of the substitutions in other viral components appear to be unique to A/equine/Ankara/1/2013. Of the 13 amino acid substitutions in the polymerase complex, S107N (PB2), T221A (PB1), K158R and M210T (PA) are highly conserved in European FC2 viruses since 2009/2010, and D27N and A100T (PA) are present in A/equine/Perthshire/3/2009, A/equine/East Renfrewshire/2/2011, A/equine/Cambremer/1/2012 and A/equine/Saone et Loire/1/2015, but the other seven substitutions appear to be unique to A/equine/Ankara/1/2013. Of the five substitutions in the NS1, only one is highly conserved since 2009 and the remaining four appear to be unique to strain. However, the other non‐structural protein PB1‐F2 substitution D50V is highly conserved since 2010 and R75H is present in A/equine/Cambremer/1/2012 and A/equine/Saone et Loire/1/2015.

## 4 DISCUSSION

The EI genome is subject to a slower rate of evolution than other mammalian influenza viruses; however, mutability as measured by the ratio of non‐synonymous to synonymous nucleotide mutations (dN/dS) was not significantly different for equine,^31^ avian^32^ or swine influenza viruses.^33^ Therefore, the genome of EIV has the potential to experience significant antigenic drift in a similar but decelerated time frame to avian and swine influenza.^31^ Thus, continuous epidemiological and phylogenetic surveillance and reporting of outbreaks in vaccinated populations are essential to determine when vaccines require updating. The aims of this study were (i) to genetically and antigenically analyse the virus responsible for the first recorded outbreak of EI in Turkey, and (ii) to ascertain whether there was any indication that OIE recommendations on vaccine strain composition required updating.

Laboratory confirmation of EIV was achieved successfully both by qRT‐PCR and the rapid detection kit Directigen EZ Flu A + B^™^ with the exception of the nasopharyngeal swab which had the highest C_*t*_ value and tested negative by Directigen. This result corroborates work undertaken by Galvin et al (2014) and illustrates that Directigen is a sensitive EIV antigen detection kit which is useful for on‐site EIV detection and/or initial screening in quarantine situations.^34^ The qRT‐PCR results were obtained within 24 hours of sample receipt by the laboratory, and the OIE received a disease alert from Turkey the following day.

Single radial haemolysis and the IDScreen influenza A antibody ELISA both yielded negative results, thus demonstrating the absence of antibodies to HA and NP, respectively, and confirming the susceptibility of horses to EIV infection.^35^, ^36^ The seronegative acutely affected horses housed at the Turkish racecourse were unvaccinated as they were at the early stages of training. The other horses had received a booster vaccine 3 months before the outbreak. Vaccination with epidemiologically relevant EIV strains activates the adaptive immune response and provides clinical and virological protection against potential future EIV infection.^35^ Strategic vaccination preceding travel and participation in racing events optimises antibody levels during periods of heightened risk of exposure.^37^ However, periods of immunological susceptibility and the mixing of naïve horses with vaccinated horses can lead to outbreaks of influenza.^38^, ^39^ It is preferable to apply mandatory vaccination to all horses that enter a facility irrespective of their activities as seronegative horses are frequently the index case in an outbreak.^39^ The amount of virus they shed coupled with frequent coughing represents a threat to their vaccinated companions, who may then become infected and succumb to influenza. However, at Ankara racetrack the affected horses were effectively quarantined, thus reducing the spread of the virus. Furthermore although the vaccine routinely used at Ankara had not been updated in line with the OIE recommendations, the regular vaccination of the racing horses at six monthly intervals appeared to have induced a protective immunity as only minimal clinical signs, that is transient pyrexia of 38.6‐40°C and slight serous nasal discharge, were observed in these horses. This was in contrast to the younger unvaccinated horses that were described as having a hacking cough.

Based on antigenic characterisation and phylogenetic analysis, the causative virus of this EI outbreak in Turkey is of the FC2 sublineage. More specifically, sequence analysis of the HA indicated that A/equine/Ankara/1/2013 clustered with viruses from the 179 group which includes viruses from Italy, Germany, France, Sweden and the UK. Thus, the virus probably spread to Turkey as a result of the relocation of horses from Europe. Back et al^13^ analysed a Swedish outbreak that occurred in 2011 where FC1 and FC2 cocirculated and reported that the index case of FC2 was transported to Sweden from Spain through the Netherlands.^13^ Other studies, based primarily on HA1 analysis, have also shown the ease with which EIV can spread as a result of transport of horses across Europe,^5^ specifically within Ireland,^24^ the UK^30^ and France.^40^, ^41^

Vaccinated horses arriving at the racecourse have the potential to shed virus without displaying clinical signs of infection. In Hong Kong in 1992, an outbreak of EI was attributed to the importation of vaccinated horses from the UK and Ireland.^42^ The incursion resulted in approximately 1000 vaccinated racehorses developing clinical signs and the postponement of seven race meetings over a period of 32 days. In contrast, this outbreak at Ankara racetrack was reported as resolved to the OIE on the 24^th^ August and although temporary movement restrictions were imposed, no race meetings were postponed.

Traditionally, genetic characterisation of EI has focussed on the HA to the exclusion of other genes. However, in recent times NA sequencing has become an integral part of the process and there is a growing realisation that genome‐scale analysis will broaden our understanding of the evolution of EIV and may assist in future control of disease. Whole‐genome sequencing of A/equine/Ankara/1/2013 was undertaken. When compared with the OIE recommended FC2 vaccine strain A/equine/Richmond/1/2007, the amino acid sequence of the surface glycoproteins HA and NA of A/equine/Ankara/1/2013 had substitutions shared by other FC2 viruses that evolved and in some cases become fixed, between 2007 and 2013. One unique substitution I214T was observed close to the HA antigenic site D. Accumulation of substitutions within antibody binding sites can potentially lead to immune escape and reduced vaccine efficacy. However, antisera generated against FC2 representatives, including A/equine/Meath/2007 which has an identical HA1 and NA amino acid sequence to A/equine/Richmond/1/2007, are strongly cross‐reactive against the Turkish strain by the HI test. Thus, there was no evidence of significant antigenic drift or any necessity to modify the current OIE recommendations for the composition of EI vaccines. Furthermore, analysis of virus components in addition to the surface glycoproteins indicated that A/equine/Ankara/1/2013 is similar to the viruses circulating in European from 2009 to 2015 and in particular those in the 179 group, for example A/equine/East Renfrewshire/2/2011, A/equine/Cambremer/1/2012 and to a lesser extent, A/equine/Saone et Loire/1/2015. The greatest diversity was observed in the NS1 and the polymerase complex. The non‐structural proteins NS1, PB1‐F2, PA‐X and the polymerase complex are important determinants of virulence that are not under the same evolutionary pressure as the surface glycoproteins. The polymerase has no proofreading ability, and the associated errors are essential to virus evolution including mutations in the HA and NA that impact on vaccine efficacy. NS1 is an interferon antagonist capable of suppressing host innate immune response, and EI viruses with a truncated NS1 have shown to be safe and effective modified live virus vaccine candidates.^43^, ^44^ The NS1 protein of EIV has evolved in a parallel fashion to the HA and in a phylogenetic analysis FC1 and FC2 NS1 amino acid sequences cluster separately.^45^ The A/equine/Ankara/1/2013 NS1 amino acid sequence is closely related to the NS1 sequences of viruses in the 179 group and does not have some of the substitutions that are highly conserved in viruses of the 144 group (Fig. S3.11). Mutations within the functional domain of NS1 protein have been associated with increased virulence in mice, humans and chickens.^46^ The significance of unique substitutions observed in the amino acid sequence of A/equine/Ankara/1/2013 is currently undetermined; however, they are located within NS1′s functional domains: 2 in the N‐terminal RNA‐binding domain (amino acid residues: 1‐73) and 2 in the C‐terminal effector domain (amino acid residues 74‐230).^47^

The PB1‐F2 triggers apoptosis of host immune cells and leads to decreased antigen presentation and diminished adaptive immune response.^48^, ^49^ It has been suggested that in addition to other non‐human influenza strains such as those of birds and pigs, EIV strains may act as donors of virulent PB1‐F2 to humans indicating a need for ongoing surveillance for genetic markers of virulence to improve pandemic preparedness.^50^ A recent study of French EIV strains highlighted a potential pro‐inflammatory motif in PB1‐F2 of A/equine/Belfond/6‐2/2009, an FC1 strain.^51^ However, the A/equine/Ankara/1/2013 PB1‐F2 sequence is similar to those of FC2 strains A/equine/Cambremer/1/2012 and A/equine/Saone et Loire/1/2015 which possess a non‐inflammatory motif (amino acid L62, H75 and Q79) (Fig. S3.3).

In conclusion, the outbreak of respiratory disease at the racetrack in Ankara was caused by an FC2 virus closely related to those circulating in Europe. There was no indication that the virus was particularly virulent and the majority of horses recovered clinically within 5 days. However, such outbreaks are disruptive to training schedules and the temporary ban on movement from the track prevented horses from racing elsewhere and reduced potential earnings. Young unvaccinated horses were a major contributor to the outbreak and emphasised the need for greater focus on herd immunity and the vaccination of all horses stabled at the track. The vaccine used, that is Equilis Prequenza, contained A/equine/Newmarket/2/93 and A/equine/South‐Africa/4/03 but had not been updated in line with OIE recommendations to contain a clade 2 virus. The antigenic characterisation with specific ferret antisera demonstrated that the A/equine/Ankara/1/2013 is significantly less closely related to A/equine/South‐Africa/4/03 a clade 1 virus than to other clade 2 viruses. Thus, it is likely that had the vaccine contained a clade 2 virus the effects of this virus incursion would have been reduced. Finally, there was no evidence of significant antigenic drift of A/equine/Ankara/1/2013 from other clade 2 viruses or any requirement to modify OIE recommendations for vaccine strain composition. However, detailed investigation of such outbreaks and characterisation of the causative strain is essential for the selection of appropriate vaccine strains and avoidance of disruption to the racing industry.
