# Supplementary material for: Whole‐genome sequencing and antigenic analysis of the first equine influenza virus identified in Turkey
Source: Influenza Other Respir Viruses. 2018 Feb 8;12(3):374–82. doi: 10.1111/irv.12485 (PMC5907808; doi:10.1111/irv.12485)
Supplement: Supplementary file 1 [file IRV-12-374-s001.docx]

**Supplementary figure 1: Alignment of the HA1 amino acid sequences.**

Alignment of the predicted HA1 amino acid sequences of Florida sublineage clade 2 strains isolated between 2007-2015 (including A/equine/Ankara/1/2013) against OIE reference strain A/equine/Richmond/1/2007. The following groups are highlighted, red: 144 group, green:179 group and blue: Asian. Amino acid identity is represented with a dot.

10 20 30 40 50 60 70 80 90 100

....|....|....|....|....|....|....|....|....|....|....|....|....|....|....|....|....|....|....|....|

**Richmond/1/2007**  **SQNPISNNNTATLCLGHHAVANGTLVKTISDDQIEVTNATELVQSISMGKICNNSYRILDGRNCTLIDAMLGDPHCDVFQYENWDLFIERSSAFSNCYPY**

**Spain/1/2007**  **....................................................................................................**

**Meath/1/2007**  **....................................................................................................**

**Pulawy/1/2008**  **....................................................................................................**

**Down/1/2008**  **........S...........................................................................................**

**Spain/1/2009**  **....................................................................................................**

**Perthshire/3/2009**  **.................................................................................G..................**

**Yorkshire/3/2009**  **....................................................................................................**

**Hamburg/2010**  **....................................................................................................**

**Shropshire/2010**  **....................................................................................................**

**Marcy-l'Etoile/1/2010**  **.................................N...............................F..................................**

**Kildare/2/2010**  **....................................................................................................**

**Tiaret/1/2011**  **....................................................................................................**

**Neuville-Pres-Sees/1/2011**  **....................................................................................................**

**Kilkenny/2011**  **....................................................................................................**

**Carlow/2011**  **....................................................................................................**

**Devon/1/2011**  **....................................................................................................**

**Kildare/2/2012**  **....................................................................................................**

**County Durham/2/2012**  **....................................................................................................**

**Northamptonshire/1/2013**  **....................................................................................................**

**Gironde/1/2014**  **....................................................................................................**

**Buckinghamshire/1/2014**  **....................................................................................................**

**Kent/1/2015**  **....................................................................................................**

**East Sussex/1/2015**  **....................................................................................................**

**Ittlingen/1/2011**  **....................................................................................................**

**East Renfrewshire/2/2011**  **....................................................................................................**

**Sweden/VIR160172/2011**  **..K.........F.......................................................................................**

**Lichtenfeld/1/2012**  **....................................................................................................**

**Cambremer/1/2012**  **.............................................T......................................................**

**Ankara/1/2013**  **....................................................................................................**

**Rome/1/2014**  **.............................................T......................................................**

**N. Rhine Westphalia/1/2014** **.............................................T......................................................**

**Saone-et-Loire/1/2015**  **.............................................T......................................................**

**Mongolia/3/2011**  **....................................................................................................**

**South Kazakhstan/236/2012 ....................................................................................................**

**Heilongjiang/SS1/2013**  **....................................................................................................**

**Supplementary Figure 1– HA1 amino acid alignment**

110 120 130 140 150 160 170 180 190 200

....|....|....|....|....|....|....|....|....|....|....|....|....|....|....|....|....|....|....|....|

**Richmond/1/2007**  **DIPDYASLRSIVASSGTLEFTAEGFTWTGVTQNGRSGACKRGSADSFFSRLNWLTKSGNSYPTLNVTMPNNKNFDKLYIWGIHHPSSNQEQTKLYIQESG**

**Spain/1/2007**  **....................................................................................................**

**Meath/1/2007**  **....................................................................................................**

**Pulawy/1/2008**  **..................................G.................................................................**

**Down/1/2008**  **....................................................................................................**

**Spain/1/2009**  **....................................................................................................**

**Perthshire/3/2009**  **....................................................................................................**

**Yorkshire/3/2009**  **....................................................................................................**

**Hamburg/2010**  **..L........I........................................................................................**

**Shropshire/2010**  **..L........I........................................................................................**

**Marcy-l'Etoile/1/2010**  **..L........I........................................................................................**

**Kildare/2/2010**  **..L........I........................................................................................**

**Tiaret/1/2011**  **..L........I........................................................................................**

**Neuville-Pres-Sees/1/2011**  **..L........I........................................................................................**

**Kilkenny/2011**  **..L........I........................................................................................**

**Carlow/2011**  **..L........I...............................V........................H...............................**

**Devon/1/2011**  **..L........I...............................V........................................................**

**Kildare/2/2012**  **..L........I...............................V........................................................**

**County Durham/2/2012**  **..L........I...............................V........................................................**

**Northamptonshire/1/2013**  **..L........I...............................V........................................................**

**Gironde/1/2014**  **..L........I...............................V........................................................**

**Buckinghamshire/1/2014**  **..L........I...............................V........................................................**

**Kent/1/2015**  **..L........I...............................V........................................................**

**East Sussex/1/2015**  **..L........I...............................V...............................................K........**

**Ittlingen/1/2011**  **..L........I..................................................................V.....................**

**East Renfrewshire/2/2011**  **..L........I..................................................................V.....................**

**Sweden/VIR160172/2011**  **..L........I..................................................................V.....................**

**Lichtenfeld/1/2012**  **...........I..................................................................V.....................**

**Cambremer/1/2012**  **..L........I......................X...........................................V............K........**

**Ankara/1/2013**  **..L........I..................................................................V.....................**

**Rome/1/2014**  **..L........I..................................................................V............K........**

**N. Rhine Westphalia/1/2014** **..L........I..................................................................V............K........**

**Saone-et-Loire/1/2015**  **..L........I..................N...K........T..................................V............K....R...**

**Mongolia/3/2011**  **...........................................T.....................................................G..**

**South Kazakhstan/236/2012**  **...........................................T.....................................................G..**

**Heilongjiang/SS1/2013**  **...........................................T.....................................................G..**

**Supplementary Figure 1 – HA1 amino acid alignment**

210 220 230 240 250 260 270 280 290 300

....|....|....|....|....|....|....|....|....|....|....|....|....|....|....|....|....|....|....|....|

**Richmond/1/2007**  **RVTVSTKRSQQTIIPNIGSRPWVRGQSGRISIYWTIVKPGDILMINSNGNLVAPRGYFKLKTGKSSVMRSDVPIDICVSECITPNGSISNEKPFQNVNKV**

**Spain/1/2007**  **..........................................................................................D.........**

**Meath/1/2007**  **....................................................................................................**

**Pulawy/1/2008**  **..........................................................................................D.........**

**Down/1/2008**  **..........................................................................................D.........**

**Spain/1/2009**  **..........................................................................................D.........**

**Perthshire/3/2009**  **..........................................................................................D.........**

**Yorkshire/3/2009**  **........N.................................................................................D.........**

**Hamburg/2010**  **..........................................................................................D.........**

**Shropshire/2010**  **..........................................................................................D.........**

**Marcy-l'Etoile/1/2010**  **..........................................................................................D.........**

**Kildare/2/2010**  **..........................................................................................D.........**

**Tiaret/1/2011**  **..........................................................................................D.........**

**Neuville-Pres-Sees/1/2011**  **..........................................................................................D.........**

**Kilkenny/2011**  **.........................................T................................................D.........**

**Carlow/2011**  **..........................................................................................D.........**

**Devon/1/2011**  **..........................................................................................D.........**

**Kildare/2/2012**  **..........................................................................................D.........**

**County Durham/2/2012**  **..........................................................................................D.........**

**Northamptonshire/1/2013**  **..........................................................................................D........I**

**Gironde/1/2014**  **..........................................................................................D........I**

**Buckinghamshire/1/2014**  **..........................................................................................D........I**

**Kent/1/2015**  **..........................................................................................D........F**

**East Sussex/1/2015**  **..................................................................I.......................D........I**

**Ittlingen/1/2011**  **..........................................................................................D.........**

**East Renfrewshire/2/2011**  **..........................................................................................D.........**

**Sweden/VIR160172/2011**  **..........................................................................................D.........**

**Lichtenfeld/1/2012**  **...............................M..........................................................D.........**

**Cambremer/1/2012**  **..........................................................................................D.........**

**Ankara/1/2013**  **.............T............................................................................D.........**

**Rome/1/2014**  **.................................................................................V........D.........**

**N. Rhine Westphalia/1/2014** **.................................................................................V........D.........**

**Saone-et-Loire/1/2015**  **.................................................................................V........D.........**

**Mongolia/3/2011**  **..........................................................................................D.........**

**South Kazakhstan/236/2012**  **..................................................................A.......................D.........**

**Heilongjiang/SS1/2013**  **...........................R...........V..................................................D.........**

**Supplementary Figure 1 – HA1 amino acid alignment**

310 320

....|....|....|....|....|....

**Richmond/1/2007**  **TYGKCPKYIRQNTLKLATGMRNVPEKQIR**

**Spain/1/2007**  **.............................**

**Meath/1/2007**  **.............................**

**Pulawy/1/2008**  **.............................**

**Down/1/2008**  **.............................**

**Spain/1/2009**  **.............................**

**Perthshire/3/2009**  **.............................**

**Yorkshire/3/2009**  **.............................**

**Hamburg/2010**  **.............................**

**Shropshire/2010**  **.............................**

**Marcy-l'Etoile/1/2010**  **.............................**

**Kildare/2/2010**  **.............................**

**Tiaret/1/2011**  **.............................**

**Neuville-Pres-Sees/1/2011**  **.............................**

**Kilkenny/2011**  **.............................**

**Carlow/2011**  **.............................**

**Devon/1/2011**  **.............................**

**Kildare/2/2012**  **.............................**

**County Durham/2/2012**  **.............................**

**Northamptonshire/1/2013**  **.............................**

**Gironde/1/2014**  **.............................**

**Buckinghamshire/1/2014**  **.............................**

**Kent/1/2015**  **.............................**

**East Sussex/1/2015**  **.............................**

**Ittlingen/1/2011**  **.............................**

**East Renfrewshire/2/2011**  **.............................**

**Sweden/VIR160172/2011**  **.............................**

**Lichtenfeld/1/2012**  **.............................**

**Cambremer/1/2012**  **.............................**

**Ankara/1/2013**  **.............................**

**Rome/1/2014**  **.............................**

**N. Rhine Westphalia/1/2014** **.............................**

**Saone-et-Loire/1/2015**  **....................K........**

**Mongolia/3/2011**  **.............................**

**South Kazakhstan/236/2012**  **.............................**

**Heilongjiang/SS1/2013**  **.............................**

**Supplementary figure 2: Alignment of the NA amino acid sequences.**

Alignment of the predicted NA amino acid sequences of Florida sublineage clade 2 strains isolated between 2007-2015 (including A/equine/Ankara/1/2013) against OIE reference strain A/equine/Richmond/1/2007. The following groups are highlighted, red: 144 group, green:179 group and blue: Asian. Amino acid identity is represented with a dot.

10 20 30 40 50 60 70 80 90 100

....|....|....|....|....|....|....|....|....|....|....|....|....|....|....|....|....|....|....|....|

**Richmond/1/2007**  **MNPNQKIITIGSASLGILIINVILHVVSIIVTVLVLNNNETGLNCKGTIIREYNETVRVEKITQWHNTSAIKYIERPPNEYYMNNTEPLCEAQGFAPFSK**

**Spain/1/2007**  **....................................................................................................**

**Perthshire/3/2009**  **.......MA..F......................A....R.D.......................Y...T.......S......................**

**Yorkshire/3/2009**  **...........F........................................................................................**

**Shropshire/2010**  **....................................................................................................**

**Neuville-Pres-Sees/1/2011** **....................................................................................................**

**Devon/1/2011**  **....................................................................................................**

**Worcestershire/1/2012**  **........................N...........................................................................**

**County Durham/2/2012**  **........................N...........................................................................**

**Northamptonshire/1/2013**  **............T...........N...........................................................................**

**Northamptonshire/5/2013**  **........................N..................S........................................................**

**Shropshire/7/2013**  **........................N................D.S........................................................**

**Lanarkshire/1/2013**  **........................N..................S........................................................**

**Gironde/1/2014**  **........................N................C.....M....................................................**

**Buckinghamshire/1/2014**  **........................N................C..........................................................**

**Kent/1/2015**  **........................N................C..........................................................**

**East Sussex/1/2015**  **........................N................C..........................................................**

**East Renfrewshire/2/2011**  **....................................................................................................**

**Sweden/VIR160172/2011**  **.........................I..........................................................................**

**Lichtenfeld/1/2012**  **.....................................Y..............................................................**

**Cambremer/1/2012**  **.....................I..............................................................................**

**Ankara/1/2013**  **..............................................R.....................................................**

**Saone-et-Loire/1/2015**  **....................................................................................................**

**South Kazakhstan/236/2012** **.....................I.......V......................................................................**

**Supplementary Figure 2- NA amino acid alignment**

110 120 130 140 150 160 170 180 190 200

....|....|....|....|....|....|....|....|....|....|....|....|....|....|....|....|....|....|....|....|

**Richmond/1/2007**  **DNGIRIGSRGHVFVIREPFVSCSPSECRTFFLTQGSLLNDKHSNGTVKDRSPYRTLMSVKIGQSPNVYQARFESVAWSATACHDGKKWMTIGVTGPDNQA**

**Spain/1/2007**  **.................................................Q.............................A....................**

**Perthshire/3/2009**  **..........................................................................................V.........**

**Yorkshire/3/2009**  **........K...........................................................................................**

**Shropshire/2010**  **........K...........................................................................................**

**Neuville-Pres-Sees/1/2011** **........K...........................................................................................**

**Devon/1/2011**  **........K...........................................................................................**

**Worcestershire/1/2012**  **........K...........................................................................................**

**County Durham/2/2012**  **........K...........................................................................................**

**Northamptonshire/1/2013**  **........K...........................................................................................**

**Northamptonshire/5/2013**  **........K...........................................................................................**

**Shropshire/7/2013**  **........K...........................................................................................**

**Lanarkshire/1/2013**  **........K...........................................................................................**

**Gironde/1/2014**  **........K...........................................................................................**

**Buckinghamshire/1/2014**  **........K...........................................................................................**

**Kent/1/2015**  **........K...........................................................................................**

**East Sussex/1/2015**  **........K...........................................................................................**

**East Renfrewshire/2/2011**  **........K...........................................................................................**

**Sweden/VIR160172/2011**  **........K...........................................................................................**

**Lichtenfeld/1/2012**  **........K...........................................................................................**

**Cambremer/1/2012**  **....................................................................................................**

**Ankara/1/2013**  **........K...........................................................................................**

**Saone-et-Loire/1/2015**  **........K...........................................................................................**

**South Kazakhstan/236/2012** **....................................................................................................**

**Supplementary Figure 2- NA amino acid alignment**

210 220 230 240 250 260 270 280 290 300

....|....|....|....|....|....|....|....|....|....|....|....|....|....|....|....|....|....|....|....|

**Richmond/1/2007**  **IAVVNYGGVPVDIINSWAGDILRTQESSCTCIKGNCYWVMTDGPANRQAKYRIFKAKDGRVIGQTDISFNGGHIEECSCYPNEGKVECICRDNWTGTNRP**

**Spain/1/2007**  **.................................................E.G................................................**

**Perthshire/3/2009**  **..................................D.................................................................**

**Yorkshire/3/2009**  **....................................................................................................**

**Shropshire/2010**  **....................................................................................................**

**Neuville-Pres-Sees/1/2011** **....................................................................................................**

**Devon/1/2011**  **...................N................................................................................**

**Worcestershire/1/2012**  **....................................................................................................**

**County Durham/2/2012**  **....................................................................................................**

**Northamptonshire/1/2013**  **....................................................................................................**

**Northamptonshire/5/2013**  **....................................................................................................**

**Shropshire/7/2013**  **....................................................................................................**

**Lanarkshire/1/2013**  **....................................................................................................**

**Gironde/1/2014**  **....................................................................................................**

**Buckinghamshire/1/2014**  **....................................................................................................**

**Kent/1/2015**  **....................................................................................................**

**East Sussex/1/2015**  **................................................................I...................................**

**East Renfrewshire/2/2011**  **........F...........................................................................................**

**Sweden/VIR160172/2011**  **....................................................................................................**

**Lichtenfeld/1/2012**  **....................................................................................................**

**Cambremer/1/2012**  **....................................................................................................**

**Ankara/1/2013**  **....................................................................................................**

**Saone-et-Loire/1/2015**  **....................................................................................................**

**South Kazakhstan/236/2012** **........I........E..................................................................................**

**Supplementary Figure 2 - NA amino acid alignment**

310 320 330 340 350 360 370 380 390 400

....|....|....|....|....|....|....|....|....|....|....|....|....|....|....|....|....|....|....|....|

**Richmond/1/2007**  **ILVISSDLSYTVGYLCAGIPTDTPRGEDSQFTGSCTSPLGNKGYGVKGFGFRQGTDVWAGRTISRTSRSGFEIIKIRNGWTQNSKDQIRRQVIIDDPNWS**

**Spain/1/2007**  **.........................................R..........................................................**

**Perthshire/3/2009**  **....................................N...............................................................**

**Yorkshire/3/2009**  **....................................................................................................**

**Shropshire/2010**  **....................................................................................................**

**Neuville-Pres-Sees/1/2011** **....................................................................................................**

**Devon/1/2011**  **....................................................................................................**

**Worcestershire/1/2012**  **....................................................................................................**

**County Durham/2/2012**  **....................................................................................................**

**Northamptonshire/1/2013**  **....................................................................................................**

**Northamptonshire/5/2013**  **....................................................................................................**

**Shropshire/7/2013**  **....................................................................................................**

**Lanarkshire/1/2013**  **....................................................................................................**

**Gironde/1/2014**  **....................................................................................................**

**Buckinghamshire/1/2014**  **....................................................................................................**

**Kent/1/2015**  **....................................................................................................**

**East Sussex/1/2015**  **..........................................................V.........................................**

**East Renfrewshire/2/2011**  **................................................................................I...................**

**Sweden/VIR160172/2011**  **....................................................................................................**

**Lichtenfeld/1/2012**  **................................................................................................S...**

**Cambremer/1/2012**  **...........................................................................V...................G....**

**Ankara/1/2013**  **................................................................................I...................**

**Saone-et-Loire/1/2015**  **................................................................................I...................**

**South Kazakhstan/236/2012** **..........................................R.....................................................Q...**

**Supplementary Figure 2 - NA amino acid alignment**

410 420 430 440 450 460 470

....|....|....|....|....|....|....|....|....|....|....|....|....|....|

**Richmond/1/2007**  **GYSGSFTLPIELTKKGCLVPCFWVEMIRGKPEETTIWTSSSSIVMCGVDHKIASWSWHDGAILPFDIDKM**

**Spain/1/2007**  **.........V............................................................**

**Perthshire/3/2009**  **.........V.....E......................................................**

**Yorkshire/3/2009**  **.........V....R.......................................................**

**Shropshire/2010**  **.........V....R..................S....................................**

**Neuville-Pres-Sees/1/2011** **.........V....R..................S....................................**

**Devon/1/2011**  **.........V....R..................S....................................**

**Worcestershire/1/2012**  **.........V....R..................S....................................**

**County Durham/2/2012**  **.........V....R..................S....................................**

**Northamptonshire/1/2013**  **.........V....R..................S....................................**

**Northamptonshire/5/2013**  **.........V....R..................S....................................**

**Shropshire/7/2013**  **.........V....R..................S....................................**

**Lanarkshire/1/2013**  **.........V....R..................S....................................**

**Gironde/1/2014**  **.........V....R..................S....................................**

**Buckinghamshire/1/2014**  **.........V....R..................S....................................**

**Kent/1/2015**  **.........V....R..................S....................................**

**East Sussex/1/2015**  **.........V....R..................S....................................**

**East Renfrewshire/2/2011**  **.........V....R..................S...................N................**

**Sweden/VIR160172/2011**  **.........V....R..................S.........M..........----------------**

**Lichtenfeld/1/2012**  **..............R..................S....................................**

**Cambremer/1/2012**  **.........V........................................E...................**

**Ankara/1/2013**  **.........V....R..................S....................................**

**Saone-et-Loire/1/2015**  **.........V.......................S.................V..................**

**South Kazakhstan/236/2012** **.........V....................................................--------**
