# Supplementary material for: Whole‐genome sequencing and antigenic analysis of the first equine influenza virus identified in Turkey
Source: Influenza Other Respir Viruses. 2018 Feb 8;12(3):374–82. doi: 10.1111/irv.12485 (PMC5907808; doi:10.1111/irv.12485)
Supplement: Supplementary file 2 [file IRV-12-374-s002.docx]

**Supplementary figures 3.1- 3.12**

Amino acid alignment of the predicted protein sequences of A/equine/Ankara/1/2013 and selected EIV Florida sublineage clade 2 strains from Europe isolated between 2009-2015 against OIE reference strain A/equine Richmond/1/2007. Amino acid identity is represented with a dot. Dashes indicate sequence at this position was unavailable. The ”144 group” is highlighted in red and the ”179 group” is highlighted in green. Sweden has been abbreviated to A/eq/Sweden/VIR160172/2013.

**3.1 PB2**

10 20 30 40 50 60 70 80 90 100 110

....|....|....|....|....|....|....|....|....|....|....|....|....|....|....|....|....|....|....|....|....|....|

**A/eq/Richmond/1/2007**  **MERIKELRDLMLQSRTREILTKTTVDHMAIIKKYTSGRQEKNPALRMKWMMAMKYPITADKRIMEMIPERNEQGQTLWSKTNDAGSDRVMVSPLAVTWWNRNGPATSTIH**

**A/eq/Perthshire/3/2009**  **..........................................................................................................N...**

**A/eq/Yorkshire/3/2009**  **..........................................................................................................N...**

**A/eq/Shropshire/2010**  **..........................................................................................................N...**

**A/eq/Neuville-Pres-Sees/1/2011** **.....................R....................................................................................N...**

**A/eq/Devon/1/2011**  **.....................R....................................................................................N...**

**A/eq/Northamptonshire/1/2013**  **.....................R....................................................................................N...**

**A/eq/Gironde/1/2014**  **........N............R....................................................................................N...**

**A/eq/Buckinghamshire/1/2014 ----------...........R....................................................................................N...**

**A/eq/Kent/1/2015 ........N............R....................................................................................N...**

**A/eq/East Sussex/1/2015**  **........N............R....................................................................................N...**

**A/eq/East Renfrewshire/2/2011**  **..........................................................................................................N...**

**A/eq/Sweden/VIR160172/2011**  **..........................................................................................................N...**

**A/eq/Cambremer/1/2012 ..........................................................................................................N...**

**A/eq/Ankara/1/2013**  **..........................................................................................................N...**

**A/eq/Saone-et-Loire/1/2015**  **..........................................................................................................N...**

120 130 140 150 160 170 180 190 200 210 220

....|....|....|....|....|....|....|....|....|....|....|....|....|....|....|....|....|....|....|....|....|....|

**A/eq/Richmond/1/2007**  **YPKVYKTYFEKVERLKHGTFGPVHFRNQVKIRRRVDVNPGHADLSAKEAQDVIMEVVFPNEVGARILTSESQLTITKEKKEELQDCKIAPLMVAYMLERELVRKTRFLPV**

**A/eq/Perthshire/3/2009**  **..............................................................................................................**

**A/eq/Yorkshire/3/2009**  **...................................................................................R..........................**

**A/eq/Shropshire/2010**  **..............................................................................................................**

**A/eq/Neuville-Pres-Sees/1/2011** ..............................................................................................................

**A/eq/Devon/1/2011**  **..............................................................................................................**

**A/eq/Northamptonshire/1/2013**  **.........................................V....................................................................**

**A/eq/Gironde/1/2014**  **.........................................V.................................................................V..**

**A/eq/Buckinghamshire/1/2014 .........................................V....................................................................**

**A/eq/Kent/1/2015 ............K............................V....................................................................**

**A/eq/East Sussex/1/2015**  **.........................................V..N.................................................................**

**A/eq/East Renfrewshire/2/2011 ..............................................................................................................**

**A/eq/Sweden/VIR160172/2011**  **..............................................................................................................**

**A/eq/Cambremer/1/2012**  **..............................................................................................................**

**A/eq/Ankara/1/2013**  **..............................................................................................................**

**A/eq/Saone-et-Loire/1/2015**  **..............................................................................................................**

230 240 250 260 270 280 290 300 310 320 330

....|....|....|....|....|....|....|....|....|....|....|....|....|....|....|....|....|....|....|....|....|....|

**A/eq/Richmond/1/2007**  **AGGTSSVYIEVLHLTQGTCWEQMYTPGGEVKNDDIDQSLIIAARNIVRRATVSADPLASLLEMCHSTQIGGIRMVDILKQNPTEEQAVDICKAAMGLRISSSFSFGGFTF**

**A/eq/Perthshire/3/2009**  **..............................................................................................................**

**A/eq/Yorkshire/3/2009**  **..............................................................................................I...............**

**A/eq/Shropshire/2010**  **..............................................................................................................**

**A/eq/Neuville-Pres-Sees/1/2011** **..............................................................................................................**

**A/eq/Devon/1/2011**  **..............................................................................................................**

**A/eq/Northamptonshire/1/2013**  **..............................................................................................................**

**A/eq/Gironde/1/2014**  **..............................................................................................................**

**A/eq/Buckinghamshire/1/2014 ..............................................................................................................**

**A/eq/Kent/1/2015 ..............................................................................................................**

**A/eq/East Sussex/1/2015**  **..............................................................................................................**

**A/eq/East Renfrewshire/2/2011**  **..........................................................................................................R...**

**A/eq/Sweden/VIR160172/2011**  **..............................................................................................................**

**A/eq/Cambremer/1/2012**  **..............................................................................................................**

**A/eq/Ankara/1/2013**  **..............................................................................................................**

**A/eq/Saone-et-Loire/1/2015**  **..............................................................................................................**

**3.1 PB2**

340 350 360 370 380 390 400 410 420 430 440

....|....|....|....|....|....|....|....|....|....|....|....|....|....|....|....|....|....|....|....|....|....|

**A/eq/Richmond/1/2007**  **KRTSGSSVKREEEMLTGNLQTLKIRVHEGYEEFTMVGRRATAILRKATRRLIQLIVSGRDEQSIAEAIIVAMVFSQEDCMIKAVRGDLNFVNRANQRLNPMHQLLRHFQK**

**A/eq/Perthshire/3/2009**  **..............................................................................................................**

**A/eq/Yorkshire/3/2009**  **..............................................................................................................**

**A/eq/Shropshire/2010**  **..............................................................................................................**

**A/eq/Neuville-Pres-Sees/1/2011** **..............................................................................................................**

**A/eq/Devon/1/2011**  **..............................................................................................................**

**A/eq/Northamptonshire/1/2013**  **..............................................................................................................**

**A/eq/Gironde/1/2014**  **..............................................................................................................**

**A/eq/Buckinghamshire/1/2014 ..............................................................................................................**

**A/eq/Kent/1/2015 ..............................................................................................................**

**A/eq/East Sussex/1/2015**  **.................................................K............................................................**

**A/eq/East Renfrewshire/2/2011**  **..............................................................................................................**

**A/eq/Sweden/VIR160172/2011**  **.....................................................F........................................................**

**A/eq/Cambremer/1/2012**  **..............................................................................................................**

**A/eq/Ankara/1/2013**  **..........................................................K...................................................**

**A/eq/Saone-et-Loire/1/2015**  **..............................................................................................................**

450 460 470 480 490 500 510 520 530 540 550

....|....|....|....|....|....|....|....|....|....|....|....|....|....|....|....|....|....|....|....|....|....|

**A/eq/Richmond/1/2007**  **DAKVLFQNWGIEPIDNVMGMIGILPDMTPSTEMSLRGVRVSKMGVDEYSSTERVVVSIDRFLRVRDQRGNILLSPEEVSETQGTEKLTIIYSSSMMWEINGPESVLVNTY**

**A/eq/Perthshire/3/2009**  **..............................................................................................................**

**A/eq/Yorkshire/3/2009**  **..............................................................................................................**

**A/eq/Shropshire/2010**  **..............................................................................................................**

**A/eq/Neuville-Pres-Sees/1/2011** **..............................................................................................................**

**A/eq/Devon/1/2011**  **..............................................................................................................**

**A/eq/Northamptonshire/1/2013**  **..............................................................................................................**

**A/eq/Gironde/1/2014**  **..............................................................................................................**

**A/eq/Buckinghamshire/1/2014 ..............................................................................................................**

**A/eq/Kent/1/2015 ..............................................................................................................**

**A/eq/East Sussex/1/2015**  **..............................................................................................................**

**A/eq/East Renfrewshire/2/2011**  **..............................................................................................................**

**A/eq/Sweden/VIR160172/2011**  **..............................................................................................................**

**A/eq/Cambremer/1/2012**  **..............................................................................................................**

**A/eq/Ankara/1/2013**  **..............................................................................................................**

**A/eq/Saone-et-Loire/1/2015**  **..............................................................................................................**

560 570 580 590 600 610 620 630 640 650 660

....|....|....|....|....|....|....|....|....|....|....|....|....|....|....|....|....|....|....|....|....|....|

**A/eq/Richmond/1/2007**  **QWIIRNWEIVKIQWSQDPTMLYNKIEFEPFQSLVPRATRSQYSGFVRTLFQQMRDVLGTFDTAQIIKLLPFAAAPPEQSRMQFSSLTVNVRGSGMRILVRGNSPVFNYNK**

**A/eq/Perthshire/3/2009**  **..............................................................................................................**

**A/eq/Yorkshire/3/2009**  **......................................................................................A......................R**

**A/eq/Shropshire/2010**  **..............................................................................................................**

**A/eq/Neuville-Pres-Sees/1/2011** **..............................................................................................................**

**A/eq/Devon/1/2011**  **..............................................................................................................**

**A/eq/Northamptonshire/1/2013**  **..............................................................................................................**

**A/eq/Gironde/1/2014 ..............................................................................................................**

**A/eq/Buckinghamshire/1/2014 ..............................................................................................................**

**A/eq/Kent/1/2015 ..............................................................................................................**

**A/eq/East Sussex/1/2015**  **..............................................................................................................**

**A/eq/East Renfrewshire/2/2011 ..............................................................................................................**

**A/eq/Sweden/VIR160172/2011**  **..............................................................................................................**

**A/eq/Cambremer/1/2012**  **..............................................................................................................**

**A/eq/Ankara/1/2013**  **...................................K..........................................................................**

**A/eq/Saone-et-Loire/1/2015**  **..............................................................................................................**

**3.1 PB2**

670 680 690 700 710 720 730 740 750

....|....|....|....|....|....|....|....|....|....|....|....|....|....|....|....|....|....|....|....

**A/eq/Richmond/1/2007**  **ATKRLTVLGKDAGALTEDPDEGTAGVESAVLRGFLILGKENKRYGPALSINELSKLAKGEKANVLIGQGDVVLVMKRKRDSSILTDSQTATKRIRMAIN**

**A/eq/Perthshire/3/2009**  **...................................................................................................**

**A/eq/Yorkshire/3/2009**  **...................................................................................................**

**A/eq/Shropshire/2010**  **...................................................................................................**

**A/eq/Neuville-Pres-Sees/1/2011** **.........X.........................................................................................**

**A/eq/Devon/1/2011**  **...................................................................................................**

**A/eq/Northamptonshire/1/2013**  **...................................................................................................**

**A/eq/Gironde/1/2014**  **...................................................................................................**

**A/eq/Buckinghamshire/1/2014 ...................................................................................................**

**A/eq/Kent/1/2015 ...................................................................................................**

**A/eq/East Sussex/1/2015**  **...................................................................................................**

**A/eq/East Renfrewshire/2/2011 ...................................................................................................**

**A/eq/Sweden/VIR160172/2011**  **...................................................................................................**

**A/eq/Cambremer/1/2012**  **.............V...........I.........................................................................**

**A/eq/Ankara/1/2013**  **...................................................................................................**

**A/eq/Saone-et-Loire/1/2015**  **.............V...........I.........................................................................**

**3.2 PB1**

10 20 30 40 50 60 70 80 90 100 110

....|....|....|....|....|....|....|....|....|....|....|....|....|....|....|....|....|....|....|....|....|....|

**A/eq//Richmond/1/2007**  **MDVNPTLLFLKVPAQNAISTTFPYTGDPPYSHGTGTGYTMDTVNRTHQYSEKGKWTTNTEIGAPQLNPIDGPLPEDNEPSGYAQTDCVLEAMAFLEESHPGIFENSCLET**

**A/eq/Perthshire/3/2009**  **.............................................................................................L................**

**A/eq/Yorkshire/3/2009**  **..............................................................................................................**

**A/eq/Shropshire/2010**  **..............................................................................................................**

**A/eq/Neuville-Pres-Sees/1/2011** **..............................................................................................................**

**A/eq/Devon/1/2011**  **..............................................................................................................**

**A/eq/Northamptonshire/1/2013**  **..............................................................................................................**

**A/eq/Gironde/1/2014**  **......V.I.....................................................V...............................................**

**A/eq/Buckinghamshire/1/2014 ..............................................................V...............................................**

**A/eq/Kent/1/2015 ..............................................................V...............................................**

**A/eq/East Sussex/1/2015**  **..............................................................V...............................................**

**A/eq/East Renfrewshire/2/2011 ...........M..................................................T...............................................**

**A/eq/Sweden/VIR160172/2011**  **..............................................................................................................**

**A/eq/Cambremer/1/2012**  **..............................................................................................................**

**A/eq/Ankara/1/2013**  **..........................................................................K...................................**

**A/eq/Saone-et-Loire/1/2015**  **..............................................................................................................**

120 130 140 150 160 170 180 190 200 210 220

....|....|....|....|....|....|....|....|....|....|....|....|....|....|....|....|....|....|....|....|....|....|

**A/eq//Richmond/1/2007**  **MEVIQQTRMDKLTQGRQTYDWTLNRNQPAATALANTIEVFRSNGLTSNESGRLMDFLKDVMESMNKEEMEITTHFQRKRRVRDNMTKRMVTQRTIGKKKQRLNRKSYLIR**

**A/eq/Perthshire/3/2009**  **........V.....................................................................................................**

**A/eq/Yorkshire/3/2009**  **..............................................................................................................**

**A/eq/Shropshire/2010**  **..............................................................................................................**

**A/eq/Neuville-Pres-Sees/1/2011** **.........................................................R....................................................**

**A/eq/Devon/1/2011**  **.........................................................R....................................................**

**A/eq/Northamptonshire/1/2013**  **.........................................................X....................................................**

**A/eq/Gironde/1/2014**  **.........................................................R..............A.....................................**

**A/eq/Buckinghamshire/1/2014 .........................................................R....................................................**

**A/eq/Kent/1/2015 ..............................................................................................................**

**A/eq/East Sussex/1/2015**  **.........................................................R....................................................**

**A/eq/East Renfrewshire/2/2011 ..............................................................................................................**

**A/eq/Sweden/VIR160172/2011**  **...............................................................................K..............................**

**A/eq/Cambremer/1/2012**  **..............................................................................................................**

**A/eq/Ankara/1/2013**  **..............................................................................................................**

**A/eq/Saone-et-Loire/1/2015**  **...V.....................................................................................................C....**

230 240 250 260 270 280 290 300 310 320 330

....|....|....|....|....|....|....|....|....|....|....|....|....|....|....|....|....|....|....|....|....|....|

**A/eq//Richmond/1/2007**  **TLTLNTMTKDAERGKLKRRAIATPGMQIRGFVYFVETLARRICEKLEQSGLPVGGNEKKAKLANVVRKMMTNSQDTELSFTITGDNTKWNENQNPRIFLAMITYITRNRP**

**A/eq/Perthshire/3/2009**  **............................................................................................................Q.**

**A/eq/Yorkshire/3/2009**  **..............................................................................................................**

**A/eq/Shropshire/2010**  **A.............................................................................................................**

**A/eq/Neuville-Pres-Sees/1/2011** **A.............................................................................................................**

**A/eq/Devon/1/2011**  **A.............................................................................................................**

**A/eq/Northamptonshire/1/2013**  **A.............................................................................................................**

**A/eq/Gironde/1/2014**  **A.............................................................................................................**

**A/eq/Buckinghamshire/1/2014 A.............................................................................................................**

**A/eq/Kent/1/2015 A.............................................................................................................**

**A/eq/East Sussex/1/2015**  **A.............................................................................................................**

**A/eq/East Renfrewshire/2/2011**  **A.............................................................................................................**

**A/eq/Sweden/VIR160172/2011**  **A.............................................................................................................**

**A/eq/Cambremer/1/2012**  **A.............................................................................................................**

**A/eq/Ankara/1/2013**  **A.............................................................................................................**

**A/eq/Saone-et-Loire/1/2015**  **A...........................................................................................................Q.**

**3.2 PB1**

340 350 360 370 380 390 400 410 420 430 440

....|....|....|....|....|....|....|....|....|....|....|....|....|....|....|....|....|....|....|....|....|....|

**A/eq/Richmond/1/2007**  **EWFRNVLSIAPIMFSNKMARLGKGYMFESKSMKLRTQIPAEMLASIELKYFNDSTKKKIEKIRPLLVDGTASLSPGMMMGMFNMLSTVLGVSILNLGQRKYTKTTYWWDG**

**A/eq/Perthshire/3/2009**  **..............................................D...............................................................**

**A/eq/Yorkshire/3/2009**  **..............................................................................................................**

**A/eq/Shropshire/2010**  **..............................................................................................................**

**A/eq/Neuville-Pres-Sees/1/2011** **..............................................................................................................**

**A/eq/Devon/1/2011**  **..............................................................................................................**

**A/eq/Northamptonshire/1/2013**  **..................................................................................H...........................**

**A/eq/Gironde/1/2014**  **..............................................................................................................**

**A/eq/Buckinghamshire/1/2014 ..............................................................................................................**

**A/eq/Kent/1/2015 ..............................................................................................................**

**A/eq/East Sussex/1/2015**  **..............................................................................................................**

**A/eq/East Renfrewshire/2/2011 ..............................................................................................................**

**A/eq/Sweden/VIR160172/2011**  **..............................................................................................................**

**A/eq/Cambremer/1/2012**  **..............................................................................................................**

**A/eq/Ankara/1/2013**  **..............................................................................................................**

**A/eq/Saone-et-Loire/1/2015**  **...................................................................................................TN....GR...**

450 460 470 480 490 500 510 520 530 540 550

....|....|....|....|....|....|....|....|....|....|....|....|....|....|....|....|....|....|....|....|....|....|

**A/eq/Richmond/1/2007**  **LQSSDDFALIVNAPNHEGIQAGVDRFYRTCKLVGINMSKKKSYINRTGTFEFTSFFYRYGFVANFSMELPSFGVSGINESADMSIGVTVIKNNMINNDLGPATAQMALQL**

**A/eq/Perthshire/3/2009**  **..............................................................................................................**

**A/eq/Yorkshire/3/2009**  **..............................................................................................................**

**A/eq/Shropshire/2010**  **..............................................................................................................**

**A/eq/Neuville-Pres-Sees/1/2011** **..............................................................................................................**

**A/eq/Devon/1/2011**  **..............................................................................................................**

**A/eq/Northamptonshire/1/2013**  **..............................................................................................................**

**A/eq/Gironde/1/2014**  **..............................................................................................................**

**A/eq/Buckinghamshire/1/2014 ..............................................................................................................**

**A/eq/Kent/1/2015 ..............................................................................................................**

**A/eq/East Sussex/1/2015**  **..............................................................................................................**

**A/eq/East Renfrewshire/2/2011 ..............................................................................................................**

**A/eq/Sweden/VIR160172/2011**  **..............................................................................................................**

**A/eq/Cambremer/1/2012**  **..............................................................................................................**

**A/eq/Ankara/1/2013**  **...............Y..............................................................................................**

**A/eq/Saone-et-Loire/1/2015**  **.HR.VY.................N......................................................................................**

560 570 580 590 600 610 620 630 640 650 660

....|....|....|....|....|....|....|....|....|....|....|....|....|....|....|....|....|....|....|....|....|....|

**A/eq/Richmond/1/2007**  **FIKDYRYTYRCHRGDTQIQTRRSFELKKLWEQTRSKTGLLVSDGGPNLYNIRNLHIPEVCLKWELMDDDYKGRLCNPLNPFVSHKEIESVNSAVVMPAHGPAKSMEYDAV**

**A/eq/Perthshire/3/2009**  **...................................................................E..R.......................................**

**A/eq/Yorkshire/3/2009**  **..............................................................................................................**

**A/eq/Shropshire/2010**  **..............................................................................................................**

**A/eq/Neuville-Pres-Sees/1/2011** **..............................................................................................................**

**A/eq/Devon/1/2011**  **..............................................................................................................**

**A/eq/Northamptonshire/1/2013**  **..............................................................................................................**

**A/eq/Gironde/1/2014**  **..............................................................................................................**

**A/eq/Buckinghamshire/1/2014 ..............................................................................................................**

**A/eq/Kent/1/2015 ...........................................................................................N..................**

**A/eq/East Sussex/1/2015**  **..............................................................................................................**

**A/eq/East Renfrewshire/2/2011 ..............................................................................................................**

**A/eq/Sweden/VIR160172/2011**  **.................................Q.................................E..R......................I................**

**A/eq/Cambremer/1/2012**  **..............................................................................................................**

**A/eq/Ankara/1/2013**  **..............................................................................................................**

**A/eq/Saone-et-Loire/1/2015**  **..............................................................................................................**

**3.2 PB1**

670 680 690 700 710 720 730 740 750

....|....|....|....|....|....|....|....|....|....|....|....|....|....|....|....|....|....|....|..

**A/eq/Richmond/1/2007**  **ATTHSWIPKRNRSILNTSQRGILEDEQMYQKCCNLFEKFFPSSSYRRPVGISSMVEAMVSRARIDARIDFESGRIKKDEFAEIMKICSTIEELRRQK**

**A/eq/Perthshire/3/2009**  **.................................................................................................**

**A/eq/Yorkshire/3/2009**  **.................................................................................................**

**A/eq/Shropshire/2010**  **.................................................................................................**

**A/eq/Neuville-Pres-Sees/1/2011** **.................................................................................................**

**A/eq/Devon/1/2011**  **.................................................................................................**

**A/eq/Northamptonshire/1/2013**  **.................................................................................................**

**A/eq/Gironde/1/2014**  **.................................................................................................**

**A/eq/Buckinghamshire/1/2014 .................................................................................................**

**A/eq/Kent/1/2015 .................................................................................................**

**A/eq/East Sussex/1/2015**  **.................................................................................................**

**A/eq/East Renfrewshire/2/2011 .................................................................................................**

**A/eq/Sweden/VIR160172/2011**  **.................................................................................................**

**A/eq/Cambremer/1/2012**  **.................................................................................................**

**A/eq/Ankara/1/2013**  **.................................................................................................**

**A/eq/Saone-et-Loire/1/2015**  **.................................................................................................**

**3.3 PB1-F2**

10 20 30 40 50 60 70 80 90

....|....|....|....|....|....|....|....|....|....|....|....|....|....|....|....|....|....|

**A/eq/Richmond/1/2007**  **MEQEQDTPWILSTEHTNIQKRGNGQQTLRLEHHNLIQSMDHFLKTMNQVDTPKQIVYWKQWLSLKNPIPESLKIRVLKQWR---------**

**A/eq/Perthshire/3/2009**  **...G................K............................G............Y...............R..---------**

**A/eq/Yorkshire/3/2009**  **.................................................G...............................---------**

**A/eq/Shropshire/2010**  **.................................................V...............................---------**

**A/eq/Neuville-Pres-Sees/1/2011** **.................................................V...............................---------**

**A/eq/Devon/1/2011**  **.................................................V...............................---------**

**A/eq/Northamptonshire/1/2013**  **..........Q.......R..............................V...............................---------**

**A/eq/Gironde/1/2014**  **......I...Q.......R............Y.................V...............................---------**

**A/eq/Buckinghamshire/1/2014**  **..........Q.......R............Y.................V...............................---------**

**A/eq/Kent/1/2015**  **..........Q.......R............Y.................V...............................---------**

**A/eq/East Sussex/1/2015**  **..........Q.......R............Y.................V...............................---------**

**A/eq/East Renfrewshire/2/2011**  **.................................................V...............................---------**

**A/eq/Sweden/VIR160172/2011**  **.................................................V...............................---------**

**A/eq/Cambremer/1/2012**  **.................................................V........................H......---------**

**A/eq/Ankara/1/2013**  **.................................................V........................H......---------**

**A/eq/Saone-et-Loire/1/2015**  **....................K............................V..................L.....H......WFSRQEWTN**

**3.4 PA**

10 20 30 40 50 60 70 80 90 100 110

....|....|....|....|....|....|....|....|....|....|....|....|....|....|....|....|....|....|....|....|....|....|

**A/eq/Richmond/1/2007**  **MEDFVRQCFNPMIVELAEKAMKEYGEDPKIETNKFAAICTHLEVCFMYSDFHFINELGESVVIDSGDPNALLKHRFEIIEGRDRTIAWTVVNSICNTTRAEKPKFLPDLY**

**A/eq/Perthshire/3/2009**  **..........................N........................................................................T..........**

**A/eq/Yorkshire/3/2009**  **..............................................................................................................**

**A/eq/Shropshire/2010**  **..............................................................................................................**

**A/eq/Neuville-Pres-Sees/1/2011** **..........................E...................................................................................**

**A/eq/Devon/1/2011**  **..........................E...................................................................................**

**A/eq/Northamptonshire/1/2013**  **..........................E.......................................................................K...........**

**A/eq/Gironde/1/2014**  **..........................E.......................................................................K...........**

**A/eq/Buckinghamshire/1/2014 ..........................E.......................................................................K...........**

**A/eq/Kent/1/2015 ..........................E............................G..........................................K...........**

**A/eq/East Sussex/1/2015**  **..........................E.......................................................................K...........**

**A/eq/East Renfrewshire/2/2011 ..........................N........................................................................T..........**

**A/eq/Sweden/VIR160172/2011**  **-.........................................................K....E.....................M........................**

**A/eq/Cambremer/1/2012**  **..........................N........................................................................T..........**

**A/eq/Ankara/1/2013**  **.....................R....N........................................................................T..........**

**A/eq/Saone-et-Loire/1/2015**  **....L.K...................N........................................................................T..........**

120 130 140 150 160 170 180 190 200 210 220

....|....|....|....|....|....|....|....|....|....|....|....|....|....|....|....|....|....|....|....|....|....|

**A/eq/Richmond/1/2007**  **DYKENRFVEIGVTRREVHIYYLEKANKIKSEKTHIHIFSFTGEEMATKADYTLDEESRARIKTRLFTIRQEMASRGLWDSFRQSERGEETIEERFEITGMMRKLANYSLP**

**A/eq/Perthshire/3/2009**  **...............................................R...................................................T..........**

**A/eq/Yorkshire/3/2009**  **...............................................R...................................................T..........**

**A/eq/Shropshire/2010**  **...............................................R...................................................T..........**

**A/eq/Neuville-Pres-Sees/1/2011** **...............................................R...................................................T..........**

**A/eq/Devon/1/2011**  **...............................................R...................................................T..........**

**A/eq/Northamptonshire/1/2013**  **...............................................R...................................................T..........**

**A/eq/Gironde/1/2014**  **...............................................R...................................................T..........**

**A/eq/Buckinghamshire/1/2014 ...............................................R...................................................T..........**

**A/eq/Kent/1/2015 ...............................................R...................................................T..........**

**A/eq/East Sussex/1/2015**  **...............................................R...................................................T..........**

**A/eq/East Renfrewshire/2/2011 ...............................................R...................................................T..........**

**A/eq/Sweden/VIR160172/2011**  **...................................................................................................T..........**

**A/eq/Cambremer/1/2012**  **...............................................R...................................................T..........**

**A/eq/Ankara/1/2013**  **...............................................R...................................................T..........**

**A/eq/Saone-et-Loire/1/2015**  **...............................................R...I...............................................T..........**

230 240 250 260 270 280 290 300 310 320 330

....|....|....|....|....|....|....|....|....|....|....|....|....|....|....|....|....|....|....|....|....|....|

**A/eq/Richmond/1/2007**  **PNFSSLENFRVYVDGFKPNGCIESKLSQMSKEVNARIEPFSKTTPRPLKMPGGPPCHQRSKFLLMDALKLSIEDPSHEGEGIPLYDAIKCMKTFFGWKEPNIVKPHEKGI**

**A/eq/Perthshire/3/2009**  **..............................................................................................................**

**A/eq/Yorkshire/3/2009**  **..............................................................................................................**

**A/eq/Shropshire/2010**  **..............................................................................................................**

**A/eq/Neuville-Pres-Sees/1/2011** **..............................................................................................................**

**A/eq/Devon/1/2011**  **..............................................................................................................**

**A/eq/Northamptonshire/1/2013**  **..............................................................................................................**

**A/eq/Gironde/1/2014**  **..............................................................................................................**

**A/eq/Buckinghamshire/1/2014 ..............................................................................................................**

**A/eq/Kent/1/2015 ........................................T.....................................................................**

**A/eq/East Sussex/1/2015**  **..............................................................................................................**

**A/eq/East Renfrewshire/2/2011 ..............................................................................................................**

**A/eq/Sweden/VIR160172/2011**  **......................................S.............................................................S.........**

**A/eq/Cambremer/1/2012**  **..............................................................................................................**

**A/eq/Ankara/1/2013**  **..............................................................................................................**

**A/eq/Saone-et-Loire/1/2015**  **...P....................................................Y.....................................................**

**3.4 PA**

340 350 360 370 380 390 400 410 420 430 440

....|....|....|....|....|....|....|....|....|....|....|....|....|....|....|....|....|....|....|....|....|....|

**A/eq/Richmond/1/2007**  **NPNYLQTWKQVLEEIQDLENEERIPKTKNMKKTSQLKWALGENMAPEKVDFEDCKDISDLKQYDSDEPETRSLASWIQSEFNKACELTDSSWIELDEIGEDVAPIEYIAS**

**A/eq/Perthshire/3/2009**  **..............................................................................................................**

**A/eq/Yorkshire/3/2009**  **......................K.......................................................................................**

**A/eq/Shropshire/2010**  **...................K..K.......................................................................................**

**A/eq/Neuville-Pres-Sees/1/2011** **......................K.......................................................................................**

**A/eq/Devon/1/2011**  **......................K.......................................................................................**

**A/eq/Northamptonshire/1/2013**  **......................K.......................................................................................**

**A/eq/Gironde/1/2014**  **......................K.........................M.............................................................**

**A/eq/Buckinghamshire/1/2014 ......................K.......................................................................................**

**A/eq/Kent/1/2015 ....................K.K.......................................................................................**

**A/eq/East Sussex/1/2015**  **......................K.......................................................................................**

**A/eq/East Renfrewshire/2/2011 ..............................................................................................................**

**A/eq/Sweden/VIR160172/2011**  **.................I............................................................N...............................**

**A/eq/Cambremer/1/2012**  **..............................................................................................................**

**A/eq/Ankara/1/2013**  **...............................................................N..............................................**

**A/eq/Saone-et-Loire/1/2015**  **..............................................................................................................**

450 460 470 480 490 500 510 520 530 540 550

....|....|....|....|....|....|....|....|....|....|....|....|....|....|....|....|....|....|....|....|....|....|

**A/eq/Richmond/1/2007**  **MRRNYFTAEISHCRATEYIMKGVYINTALLNASCATMDEFQLIPMISKCRTKEGRRKTNLYGFIIKGRSHLRNDTDVVNFVSMEFSLTDPRFEPHKWEKYCVLEIGDMLL**

**A/eq/Perthshire/3/2009**  **..............................................................................................................**

**A/eq/Yorkshire/3/2009**  **...................................K..........................................................................**

**A/eq/Shropshire/2010**  **..............................................................................................................**

**A/eq/Neuville-Pres-Sees/1/2011** **..............................................................................................................**

**A/eq/Devon/1/2011**  **..............................................................................................................**

**A/eq/Northamptonshire/1/2013**  **..............................................................................................................**

**A/eq/Gironde/1/2014**  **..............................................................................................................**

**A/eq/Buckinghamshire/1/2014 ..............................................................................................................**

**A/eq/Kent/1/2015 ..............................................................................................................**

**A/eq/East Sussex/1/2015**  **..............................................................................................................**

**A/eq/East Renfrewshire/2/2011 ..............................................................................................................**

**A/eq/Sweden/VIR160172/2011**  **........................V..........A............................V.............................................**

**A/eq/Cambremer/1/2012**  **..............................................................................................................**

**A/eq/Ankara/1/2013**  **..........................................................................................K...................**

**A/eq/Saone-et-Loire/1/2015**  **..............................................................................................................**

560 570 580 590 600 610 620 630 640 650 660

....|....|....|....|....|....|....|....|....|....|....|....|....|....|....|....|....|....|....|....|....|....|

**A/eq/Richmond/1/2007**  **RTAVGQVSRPMFLYVRTNGTSKIKMKWGMEMRRCLLQSLQQIESMIEAESSVKEKDMTKEFFENKSETWPIGESPRGVEEGSIGKVCRTLLAKSVFNSLYASPQLEGFSA**

**A/eq/Perthshire/3/2009**  **..............................................................................................................**

**A/eq/Yorkshire/3/2009**  **..............................................................................................................**

**A/eq/Shropshire/2010**  **..............................................................................................................**

**A/eq/Neuville-Pres-Sees/1/2011** **..............................................................................................................**

**A/eq/Devon/1/2011**  **..............................................................................................................**

**A/eq/Northamptonshire/1/2013**  **..............................................................................................................**

**A/eq/Gironde/1/2014**  **.....................................P........................................................................**

**A/eq/Buckinghamshire/1/2014 .....................................P........................................................................**

**A/eq/Kent/1/2015 .....................................P........................................................................**

**A/eq/East Sussex/1/2015**  **.....................................P................R.......................................................**

**A/eq/East Renfrewshire/2/2011 ..............................................................................................................**

**A/eq/Sweden/VIR160172/2011**  **...........................................................................K..................................**

**A/eq/Cambremer/1/2012**  **..............................................................................................................**

**A/eq/Ankara/1/2013**  **..............................................................................................................**

**A/eq/Saone-et-Loire/1/2015**  **..............I.......................................R.......................................................**

**3.4 PA**

670 680 690 700 710

....|....|....|....|....|....|....|....|....|....|....|.

**A/eq/Richmond/1/2007**  **ESRKLLLIVQALRDNLEPGTFDIGGLYESIEECLINDPWVLLNASWFNSFLTHALK**

**A/eq/Perthshire/3/2009**  **........................................................**

**A/eq/Yorkshire/3/2009**  **........................................................**

**A/eq/Shropshire/2010**  **........................................................**

**A/eq/Neuville-Pres-Sees/1/2011** **........................................................**

**A/eq/Devon/1/2011**  **........................................................**

**A/eq/Northamptonshire/1/2013**  **........................................................**

**A/eq/Gironde/1/2014**  **........................................................**

**A/eq/Buckinghamshire/1/2014 ........................................................**

**A/eq/Kent/1/2015 ........................................................**

**A/eq/East Sussex/1/2015**  **........................................................**

**A/eq/East Renfrewshire/2/2011 ........................................................**

**A/eq/Sweden/VIR160172/2011**  **........................................................**

**A/eq/Cambremer/1/2012**  **........................................................**

**A/eq/Ankara/1/2013**  **........................................................**

**A/eq/Saone-et-Loire/1/2015**  **........................................................**

**3.5 PAX C-TERMINAL**

200 210 220 230 240 250

....|....|....|....|....|....|....|....|....|....|....|....|..

**A/eq/Perthshire/3/2009**  **FVSPREAKRQLKKDLKSQGRCASLPITVSHRTSPALKILESMWMDSNRTAALRVSFLKCPKK**

**A/eq/Richmond/1/2007**  **--------------------..........................................**

**A/eq/Yorkshire/3/2009**  **..............................................................**

**A/eq/Shropshire/2010**  **..............................................................**

**A/eq/Neuville-Pres-Sees/1/2011** **..............................................................**

**A/eq/Devon/1/2011**  **..............................................................**

**A/eq/Northamptonshire/1/2013**  **..............................................................**

**A/eq/Gironde/1/2014**  **..............................................................**

**A/eq/Buckinghamshire/1/2014**  **..............................................................**

**A/eq/Kent/1/2015**  **.............................R................................**

**A/eq/East Sussex/1/2015**  **..............................................................**

**A/eq/East Renfrewshire/2/2011**  **..............................................................**

**A/eq/Sweden/VIR160172/2011**  **.................................................D............**

**A/eq/Cambremer/1/2012**  **..............................................................**

**A/eq/Ankara/1/2013**  **..............................................................**

**A/eq/Saone-et-Loire/1/2015**  **..............................................................**

**3.6 HA**

10 20 30 40 50 60 70 80 90 100 110

....|....|....|....|....|....|....|....|....|....|....|....|....|....|....|....|....|....|....|....|....|....|

**A/eq/Richmond/1/2007**  **SQNPISNNNTATLCLGHHAVANGTLVKTISDDQIEVTNATELVQSISMGKICNNSYRILDGRNCTLIDAMLGDPHCDVFQYENWDLFIERSSAFSNCYPYDIPDYASLRS**

**A/eq/Perthshire/3/2009**  **.................................................................................G............................**

**A/eq/Yorkshire/3/2009**  **..............................................................................................................**

**A/eq/Shropshire/2010**  **......................................................................................................L.......**

**A/eq/Neuville-Pres-Sees/1/2011** **......................................................................................................L.......**

**A/eq/Devon/1/2011**  **......................................................................................................L.......**

**A/eq/Northamptonshire/1/2013**  **......................................................................................................L.......**

**A/eq/Gironde/1/2014**  **......................................................................................................L.......**

**A/eq/Buckinghamshire/1/2014 ......................................................................................................L.......**

**A/eq/Kent/1/2015 ......................................................................................................L.......**

**A/eq/East Sussex/1/2015**  **......................................................................................................L.......**

**A/eq/East Renfrewshire/2/2011**  **......................................................................................................L.......**

**A/eq/Sweden/VIR160172/2011**  **..K.........F.........................................................................................L.......**

**A/eq/Cambremer/1/2012**  **.............................................T........................................................L.......**

**A/eq/Ankara/1/2013**  **......................................................................................................L.......**

**A/eq/Saone-et-Loire/1/2015**  **.............................................T........................................................L.......**

120 130 140 150 160 170 180 190 200 210 220

....|....|....|....|....|....|....|....|....|....|....|....|....|....|....|....|....|....|....|....|....|....|

**A/eq/Richmond/1/2007**  **IVASSGTLEFTAEGFTWTGVTQNGRSGACKRGSADSFFSRLNWLTKSGNSYPTLNVTMPNNKNFDKLYIWGIHHPSSNQEQTKLYIQESGRVTVSTKRSQQTIIPNIGSR**

**A/eq/Perthshire/3/2009**  **..............................................................................................................**

**A/eq/Yorkshire/3/2009**  **..................................................................................................N...........**

**A/eq/Shropshire/2010**  **.I............................................................................................................**

**A/eq/Neuville-Pres-Sees/1/2011** **.I............................................................................................................**

**A/eq/Devon/1/2011**  **.I...............................V............................................................................**

**A/eq/Northamptonshire/1/2013**  **.I...............................V............................................................................**

**A/eq/Gironde/1/2014**  **.I...............................V............................................................................**

**A/eq/Buckinghamshire/1/2014 .I...............................V............................................................................**

**A/eq/Kent/1/2015 .I...............................V............................................................................**

**A/eq/East Sussex/1/2015**  **.I...............................V...............................................K............................**

**A/eq/East Renfrewshire/2/2011 .I..................................................................V.........................................**

**A/eq/Sweden/VIR160172/2011**  **.I..................................................................V.........................................**

**A/eq/Cambremer/1/2012**  **.I......................X...........................................V............K............................**

**A/eq/Ankara/1/2013**  **.I..................................................................V..................................T......**

**A/eq/Saone-et-Loire/1/2015**  **.I..................N...K........T..................................V............K....R.......................**

230 240 250 260 270 280 290 300 310 320 330

....|....|....|....|....|....|....|....|....|....|....|....|....|....|....|....|....|....|....|....|....|....|

**A/eq/Richmond/1/2007**  **PWVRGQSGRISIYWTIVKPGDILMINSNGNLVAPRGYFKLKTGKSSVMRSDVPIDICVSECITPNGSISNEKPFQNVNKVTYGKCPKYIRQNTLKLATGMRNVPEKQIRG**

**A/eq/Perthshire/3/2009**  **......................................................................D.......................................**

**A/eq/Yorkshire/3/2009**  **......................................................................D.......................................**

**A/eq/Shropshire/2010**  **......................................................................D.......................................**

**A/eq/Neuville-Pres-Sees/1/2011** **......................................................................D.......................................**

**A/eq/Devon/1/2011**  **......................................................................D.......................................**

**A/eq/Northamptonshire/1/2013**  **......................................................................D........I..............................**

**A/eq/Gironde/1/2014**  **......................................................................D........I..............................**

**A/eq/Buckinghamshire/1/2014 ......................................................................D........I..............................**

**A/eq/Kent/1/2015 ......................................................................D........F..............................**

**A/eq/East Sussex/1/2015**  **..............................................I.......................D........I..............................**

**A/eq/East Renfrewshire/2/2011 ......................................................................D.......................................**

**A/eq/Sweden/VIR160172/2011**  **......................................................................D......................................-**

**A/eq/Cambremer/1/2012**  **......................................................................D.......................................**

**A/eq/Ankara/1/2013**  **......................................................................D.......................................**

**A/eq/Saone-et-Loire/1/2015**  **.............................................................V........D.............................K.........**

**3.6 HA**

340 350 360 370 380 390 400 410 420 430 440

....|....|....|....|....|....|....|....|....|....|....|....|....|....|....|....|....|....|....|....|....|....|

**A/eq/Richmond/1/2007**  **IFGAIAGFIENGWEGMVDGWYGFRYQNSEGTGQAADLKSTQTAIDQINEKLNRVIERTNEKFHQIEKEFSEVEGRIQDLEKYVEDTKIDLWSYNAELLVALENQHTIDLT**

**A/eq/Perthshire/3/2009**  **..............................................................................................................**

**A/eq/Yorkshire/3/2009**  **..............................................................................................................**

**A/eq/Shropshire/2010**  **..............................................................................................................**

**A/eq/Neuville-Pres-Sees/1/2011** **..............................................................................................................**

**A/eq/Devon/1/2011**  **..............................................................................................................**

**A/eq/Northamptonshire/1/2013**  **..............................................................................................................**

**A/eq/Gironde/1/2014**  **..............................................................................................................**

**A/eq/Buckinghamshire/1/2014 ..............................................................................................................**

**A/eq/Kent/1/2015 ..............................................................................................................**

**A/eq/East Sussex/1/2015**  **.........................................A....................................................................**

**A/eq/East Renfrewshire/2/2011 ..............................................................................................................**

**A/eq/Sweden/VIR160172/2011**  **--------------------------------------------------------------------------------------------------------------**

**A/eq/Cambremer/1/2012**  **....................................................................................................I.........**

**A/eq/Ankara/1/2013**  **..............................................................................................................**

**A/eq/Saone-et-Loire/1/2015**  **..............................................................................................................**

450 460 470 480 490 500 510 520 530 540 550

....|....|....|....|....|....|....|....|....|....|....|....|....|....|....|....|....|....|....|....|....|....|

**A/eq/Richmond/1/2007**  **DAEMNKLFEKTRRQLRENAEDMGGGCFKIYHKCDNACIGSIRNGTYDHYIYRDEALNNRFQIKGVELKSGYKDWILWISFAISCFLICVVLLGFIMWACQKGNIRCNICI**

**A/eq/Perthshire/3/2009**  **..............................................................................................................**

**A/eq/Yorkshire/3/2009**  **..............................................................................................................**

**A/eq/Shropshire/2010**  **..............................................................................................................**

**A/eq/Neuville-Pres-Sees/1/2011** **..............................................................................................................**

**A/eq/Devon/1/2011**  **..............................................................................................................**

**A/eq/Northamptonshire/1/2013**  **...........................................................................M..................................**

**A/eq/Gironde/1/2014**  **......................................................................N....M.......N................----------**

**A/eq/Buckinghamshire/1/2014 ...........................................................................M..................................**

**A/eq/Kent/1/2015 ...........................................................................M..................................**

**A/eq/East Sussex/1/2015**  **...........................................................................I..................................**

**A/eq/East Renfrewshire/2/2011 ..............................................................................................................**

**A/eq/Sweden/VIR160172/2011**  **--------------------------------------------------------------------------------------------------------------**

**A/eq/Cambremer/1/2012**  **..............................................................................................................**

**A/eq/Ankara/1/2013**  **..............................................................................................................**

**A/eq/Saone-et-Loire/1/2015**  **......................................................................................V.......................**

**3.7 NP**

10 20 30 40 50 60 70 80 90 100 110

....|....|....|....|....|....|....|....|....|....|....|....|....|....|....|....|....|....|....|....|....|....|

**A/eq/Richmond/1/2007**  **MASQGTKRSYEQMETDGERQNATEIRASVGRMVGGIGRFYVQMCTELKLNDHEGRLIQNSITIERMVLSAFDERRNKYLEEHPSAGKDPKKTGGPIYRRKDGKWMRELIL**

**A/eq/Perthshire/3/2009**  **..............................................................................................................**

**A/eq/Yorkshire/3/2009**  **..............................................................................................................**

**A/eq/Shropshire/2010**  **..............................................................................................................**

**A/eq/Neuville-Pres-Sees/1/2011** **..............................................................................................................**

**A/eq/Devon/1/2011**  **..............................................................................................................**

**A/eq/Northamptonshire/1/2013**  **..............................................................................................................**

**A/eq/Gironde/1/2014**  **..............................................................................................................**

**A/eq/Buckinghamshire/1/2014**  **..............................................................................................................**

**A/eq/Kent/1/2015**  **..............................................................................................................**

**A/eq/East Sussex/1/2015**  **..............................................................................................................**

**A/eq/East Renfrewshire/2/2011**  **..............................................................................................................**

**A/eq/Sweden/VIR160172/2011**  **...H..........................................................................................................**

**A/eq/Cambremer/1/2012**  **..............................................................................................................**

**A/eq/Ankara/1/2013**  **..............................................................................................................**

**A/eq/Saone-et-Loire/1/2015**  **...................................................................................................R..........**

120 130 140 150 160 170 180 190 200 210 220

....|....|....|....|....|....|....|....|....|....|....|....|....|....|....|....|....|....|....|....|....|....|

**A/eq/Richmond/1/2007**  **HDKEEIMRIWRQANNGEDATAGLTHIMIWHSNLNDTTYQRTRALVRTGMDPRMCSLMQGSTLPRRSGAAGAAVKGVGTMVMELIRMIKRGINDRNFWRGENGRRTRIAYE**

**A/eq/Perthshire/3/2009**  **..............................................................................................................**

**A/eq/Yorkshire/3/2009**  **..............................................................................................................**

**A/eq/Shropshire/2010**  **..............................................................................................................**

**A/eq/Neuville-Pres-Sees/1/2011** **..............................................................................................................**

**A/eq/Devon/1/2011**  **..............................................................................L...............................**

**A/eq/Northamptonshire/1/2013**  **..............................................................................I...............................**

**A/eq/Gironde/1/2014**  **Y.............................................................................................................**

**A/eq/Buckinghamshire/1/2014**  **Y.............................................................................................................**

**A/eq/Kent/1/2015**  **Y.............................................................................................................**

**A/eq/East Sussex/1/2015**  **Y.............................................................................................................**

**A/eq/East Renfrewshire/2/2011**  **..............................................................................................................**

**A/eq/Sweden/VIR160172/2011**  **..............................................................................................................**

**A/eq/Cambremer/1/2012**  **..............................................................................................................**

**A/eq/Ankara/1/2013**  **..............................................................................................................**

**A/eq/Saone-et-Loire/1/2015**  **......R.......................................................................................................**

230 240 250 260 270 280 290 300 310 320 330

....|....|....|....|....|....|....|....|....|....|....|....|....|....|....|....|....|....|....|....|....|....|

**A/eq/Richmond/1/2007**  **RMCNILKGKFQTAAQRAMMDQVREGRNPGNAEIEDLTFLARSALILRGSVAHKSCLPACVYGLAVTSGYDFEKEGYSLVGIDPFKLLQNSQIFSLIRPKENPAHKSQLVW**

**A/eq/Perthshire/3/2009**  **..............................................................................................................**

**A/eq/Yorkshire/3/2009**  **..............................................................................................................**

**A/eq/Shropshire/2010**  **..............................................................................................................**

**A/eq/Neuville-Pres-Sees/1/2011** **..............................................................................................................**

**A/eq/Devon/1/2011**  **....................................................................................R.........................**

**A/eq/Northamptonshire/1/2013**  **....................................................................................R.........................**

**A/eq/Gironde/1/2014**  **....................................................................................R.........................**

**A/eq/Buckinghamshire/1/2014**  **....................................................................................R.........................**

**A/eq/Kent/1/2015**  **....................................................................................R.........................**

**A/eq/East Sussex/1/2015**  **....................................................................................R.........................**

**A/eq/East Renfrewshire/2/2011**  **.................................................................................................L............**

**A/eq/Sweden/VIR160172/2011**  **..............................................................................................................**

**A/eq/Cambremer/1/2012**  **........................C.....................................................................................**

**A/eq/Ankara/1/2013**  **..................V...........................................................................................**

**A/eq/Saone-et-Loire/1/2015**  **..............................................................................................................**

**3.7 NP**

340 350 360 370 380 390 400 410 420 430 440

....|....|....|....|....|....|....|....|....|....|....|....|....|....|....|....|....|....|....|....|....|....|

**A/eq/Richmond/1/2007**  **MACHSAAFEDLRVLNFIRGTKVIPRGQLATRGVQIASNENMETIDSSTLELRSKYWAIRTRSGGNTSQQRASAGQISVQPTFSVQRNLPFERATIMAAFTGNTEGRTSDM**

**A/eq/Perthshire/3/2009**  **..............................................................................................................**

**A/eq/Yorkshire/3/2009**  **..............................................................................................................**

**A/eq/Shropshire/2010**  **..............................................................................................................**

**A/eq/Neuville-Pres-Sees/1/2011** **..............................................................................................................**

**A/eq/Devon/1/2011**  **..............................................................................................................**

**A/eq/Northamptonshire/1/2013**  **..............................................................................................................**

**A/eq/Gironde/1/2014**  **..............................................................................................................**

**A/eq/Buckinghamshire/1/2014**  **..............................................................................................................**

**A/eq/Kent/1/2015**  **..............................................................................................................**

**A/eq/East Sussex/1/2015**  **..............................................................................................................**

**A/eq/East Renfrewshire/2/2011**  **..............................................................................................................**

**A/eq/Sweden/VIR160172/2011**  **............................................................K.................................................**

**A/eq/Cambremer/1/2012**  **..............................................................................................................**

**A/eq/Ankara/1/2013**  **..............................................................................................................**

**A/eq/Saone-et-Loire/1/2015**  **..............................................................................................................**

450 460 470 480 490

....|....|....|....|....|....|....|....|....|....|....|...

**A/eq/Richmond/1/2007**  **RTEIIRMMESAKSEDVSFQGRGVFELSDEKATNPIVPSFDMSNEGSYFFGDNAEEFDS**

**A/eq/Perthshire/3/2009**  **..........................................................**

**A/eq/Yorkshire/3/2009**  **................................S.........................**

**A/eq/Shropshire/2010**  **................................S.........................**

**A/eq/Neuville-Pres-Sees/1/2011** **................................S.........................**

**A/eq/Devon/1/2011**  **................................S.........................**

**A/eq/Northamptonshire/1/2013**  **................................S.........................**

**A/eq/Gironde/1/2014**  **................................S.........................**

**A/eq/Buckinghamshire/1/2014**  **................................S.........................**

**A/eq/Kent/1/2015**  **................................S.........................**

**A/eq/East Sussex/1/2015**  **................................S.........................**

**A/eq/East Renfrewshire/2/2011**  **................................S.........................**

**A/eq/Sweden/VIR160172/2011**  **................................S......E..................**

**A/eq/Cambremer/1/2012**  **................................S.........................**

**A/eq/Ankara/1/2013**  **................................S.........................**

**A/eq/Saone-et-Loire/1/2015**  **................................S.........................**

**3.8 NA**

10 20 30 40 50 60 70 80 90 100 110

....|....|....|....|....|....|....|....|....|....|....|....|....|....|....|....|....|....|....|....|....|....|

**A/eq/Richmond/1/2007**  **MNPNQKIITIGSASLGILIINVILHVVSIIVTVLVLNNNETGLNCKGTIIREYNETVRVEKITQWHNTSAIKYIERPPNEYYMNNTEPLCEAQGFAPFSKDNGIRIGSRG**

**A/eq/Perthshire/3/2009**  **.......MA..F......................A....R.D.......................Y...T.......S................................**

**A/eq/Yorkshire/3/2009**  **...........F................................................................................................K.**

**A/eq/Shropshire/2010**  **............................................................................................................K.**

**A/eq/Neuville-Pres-Sees/1/2011** **............................................................................................................K.**

**A/eq/Devon/1/2011**  **............................................................................................................K.**

**A/eq/Northamptonshire/1/2013**  **............T...........N...................................................................................K.**

**A/eq/Gironde/1/2014**  **........................N................C.....M............................................................K.**

**A/eq/Buckinghamshire/1/2014 ........................N................C..................................................................K.**

**A/eq/Kent/1/2015 ........................N................C..................................................................K.**

**A/eq/East Sussex/1/2015**  **........................N................C..................................................................K.**

**A/eq/East Renfrewshire/2/2011 ............................................................................................................K.**

**A/eq/Sweden/VIR160172/2011**  **.........................I..................................................................................K.**

**A/eq/Cambremer/1/2012**  **.....................I........................................................................................**

**A/equine/Ankara/1/2013 ..............................................R.............................................................K.**

**A/eq/Saone-et-Loire/1/2015**  **............................................................................................................K.**

120 130 140 150 160 170 180 190 200 210 220

....|....|....|....|....|....|....|....|....|....|....|....|....|....|....|....|....|....|....|....|....|....|

**A/eq/Richmond/1/2007**  **HVFVIREPFVSCSPSECRTFFLTQGSLLNDKHSNGTVKDRSPYRTLMSVKIGQSPNVYQARFESVAWSATACHDGKKWMTIGVTGPDNQAIAVVNYGGVPVDIINSWAGD**

**A/eq/Perthshire/3/2009**  **................................................................................V.............................**

**A/eq/Yorkshire/3/2009**  **..............................................................................................................**

**A/eq/Shropshire/2010**  **..............................................................................................................**

**A/eq/Neuville-Pres-Sees/1/2011** **..............................................................................................................**

**A/eq/Devon/1/2011**  **.............................................................................................................N**

**A/eq/Northamptonshire/1/2013**  **..............................................................................................................**

**A/eq/Gironde/1/2014**  **..............................................................................................................**

**A/eq/Buckinghamshire/1/2014 ..............................................................................................................**

**A/eq/Kent/1/2015 ..............................................................................................................**

**A/eq/East Sussex/1/2015**  **..............................................................................................................**

**A/eq/East Renfrewshire/2/2011 ..................................................................................................F...........**

**A/eq/Sweden/VIR160172/2011**  **..............................................................................................................**

**A/eq/Cambremer/1/2012**  **..............................................................................................................**

**A/equine/Ankara/1/2013 ..............................................................................................................**

**A/eq/Saone-et-Loire/1/2015**  **..............................................................................................................**

230 240 250 260 270 280 290 300 310 320 330

....|....|....|....|....|....|....|....|....|....|....|....|....|....|....|....|....|....|....|....|....|....|

**A/eq/Richmond/1/2007**  **ILRTQESSCTCIKGNCYWVMTDGPANRQAKYRIFKAKDGRVIGQTDISFNGGHIEECSCYPNEGKVECICRDNWTGTNRPILVISSDLSYTVGYLCAGIPTDTPRGEDSQ**

**A/eq/Perthshire/3/2009**  **..............D...............................................................................................**

**A/eq/Yorkshire/3/2009**  **..............................................................................................................**

**A/eq/Shropshire/2010**  **..............................................................................................................**

**A/eq/Neuville-Pres-Sees/1/2011** **..............................................................................................................**

**A/eq/Devon/1/2011**  **..............................................................................................................**

**A/eq/Northamptonshire/1/2013**  **..............................................................................................................**

**A/eq/Gironde/1/2014**  **..............................................................................................................**

**A/eq/Buckinghamshire/1/2014 ..............................................................................................................**

**A/eq/Kent/1/2015 ..............................................................................................................**

**A/eq/East Sussex/1/2015**  **............................................I.................................................................**

**A/eq/East Renfrewshire/2/2011 ..............................................................................................................**

**A/eq/Sweden/VIR160172/2011**  **..............................................................................................................**

**A/eq/Cambremer/1/2012**  **..............................................................................................................**

**A/equine/Ankara/1/2013 ..............................................................................................................**

**A/eq/Saone-et-Loire/1/2015**  **..............................................................................................................**

**3.8 NA**

340 350 360 370 380 390 400 410 420 430 440

....|....|....|....|....|....|....|....|....|....|....|....|....|....|....|....|....|....|....|....|....|....|

**A/eq/Richmond/1/2007**  **FTGSCTSPLGNKGYGVKGFGFRQGTDVWAGRTISRTSRSGFEIIKIRNGWTQNSKDQIRRQVIIDDPNWSGYSGSFTLPIELTKKGCLVPCFWVEMIRGKPEETTIWTSS**

**A/eq/Perthshire/3/2009**  **......N........................................................................V.....E........................**

**A/eq/Yorkshire/3/2009**  **...............................................................................V....R.........................**

**A/eq/Shropshire/2010**  **...............................................................................V....R..................S......**

**A/eq/Neuville-Pres-Sees/1/2011** **...............................................................................V....R..................S......**

**A/eq/Devon/1/2011**  **...............................................................................V....R..................S......**

**A/eq/Northamptonshire/1/2013**  **...............................................................................V....R..................S......**

**A/eq/Gironde/1/2014**  **...............................................................................V....R..................S......**

**A/eq/Buckinghamshire/1/2014 ...............................................................................V....R..................S......**

**A/eq/Kent/1/2015 ...............................................................................V....R..................S......**

**A/eq/East Sussex/1/2015**  **............................V..................................................V....R..................S......**

**A/eq/East Renfrewshire/2/2011 ..................................................I............................V....R..................S......**

**A/eq/Sweden/VIR160172/2011**  **...............................................................................V....R..................S......**

**A/eq/Cambremer/1/2012**  **.............................................V...................G.............V..............................**

**A/equine/Ankara/1/2013 ..................................................I............................V....R..................S......**

**A/eq/Saone-et-Loire/1/2015**  **..................................................I............................V.......................S......**

450 460 470

....|....|....|....|....|....|

**A/eq/Richmond/1/2007**  **SSIVMCGVDHKIASWSWHDGAILPFDIDKM**

**A/eq/Perthshire/3/2009**  **..............................**

**A/eq/Yorkshire/3/2009**  **..............................**

**A/eq/Shropshire/2010**  **..............................**

**A/eq/Neuville-Pres-Sees/1/2011** **..............................**

**A/eq/Devon/1/2011**  **..............................**

**A/eq/Northamptonshire/1/2013**  **..............................**

**A/eq/Gironde/1/2014**  **..............................**

**A/eq/Buckinghamshire/1/2014 ..............................**

**A/eq/Kent/1/2015 ..............................**

**A/eq/East Sussex/1/2015**  **..............................**

**A/eq/East Renfrewshire/2/2011 .............N................**

**A/eq/Sweden/VIR160172/2011**  **...M..........----------------**

**A/eq/Cambremer/1/2012**  **..........E...................**

**A/equine/Ankara/1/2013 ..............................**

**A/eq/Saone-et-Loire/1/2015**  **...........V..................**

**3.9 M1**

10 20 30 40 50 60 70 80 90 100 110

....|....|....|....|....|....|....|....|....|....|....|....|....|....|....|....|....|....|....|....|....|....|

**A/eq/Richmond/1/2007**  **MSLLTEVETYVLSIIPSGPLKAEIAQRLEDVFAGKNTDLEALMEWLKTRPILSPLTKGILGFVFTLTVPSERGLQRRRFIQNALSGNGDPNNMDKAVKLYRKLKREITFH**

**A/eq/Perthshire/3/2009**  **..............................................................................................................**

**A/eq/Yorkshire/3/2009**  **..............................................................................................................**

**A/eq/Shropshire/2010**  **..............................................................................................................**

**A/eq/Neuville-Pres-Sees/1/2011** **..............................................................................................................**

**A/eq/Devon/1/2011**  **..............................................................................................................**

**A/eq/Northamptonshire/1/2013**  **..............................................................................................................**

**A/eq/Gironde/1/2014**  **............F......F..........................................................................................**

**A/eq/Buckinghamshire/1/2014 ..............................................................................................................**

**A/eq/Kent/1/2015 ..............................................................................................................**

**A/eq/East Sussex/1/2015**  **..............................................................................................................**

**A/eq/East Renfrewshire/2/2011 .............V................................................................................................**

**A/eq/Sweden/VIR160172/2011**  **...............................................................................V..............R...............**

**A/eq/Cambremer/1/2012**  **----.........V................................................................................................**

**A/eq/Ankara/1/2013**  **..............................................................................................................**

**A/eq/Saone-et-Loire/1/2015**  **----........FV................................................................................................**

120 130 140 150 160 170 180 190 200 210 220

....|....|....|....|....|....|....|....|....|....|....|....|....|....|....|....|....|....|....|....|....|....|

**A/eq/Richmond/1/2007**  **GAKEVALSYSTGALASCMGLIYNRMGTVTTEVAFGLVCATCEQIADSQHRSHRQMVTTTNPLIRHENRMVLASTTAKAMEQMAGSSEQAAEAMEVASKARQMVQAMRTIG**

**A/eq/Perthshire/3/2009**  **.........................................................I....................................................**

**A/eq/Yorkshire/3/2009**  **.........................................................I....................................................**

**A/eq/Shropshire/2010**  **.........................................................I....................................................**

**A/eq/Neuville-Pres-Sees/1/2011** **.........................................................I....................................................**

**A/eq/Devon/1/2011**  **.........................................................I....................................................**

**A/eq/Northamptonshire/1/2013**  **.........................................................I....................................................**

**A/eq/Gironde/1/2014**  **.........................................................I....................................................**

**A/eq/Buckinghamshire/1/2014 .........................................................I....................................................**

**A/eq/Kent/1/2015 .........................................................I....................................................**

**A/eq/East Sussex/1/2015**  **.........................................................I....................................................**

**A/eq/East Renfrewshire/2/2011 .........................................................I....................................................**

**A/eq/Sweden/VIR160172/2011**  **................................................................................................NR............**

**A/eq/Cambremer/1/2012**  **.........................................................I....................................................**

**A/eq/Ankara/1/2013**  **.........................................................I....................................................**

**A/eq/Saone-et-Loire/1/2015**  **.........................................................I....................................................**

230 240 250

....|....|....|....|....|....|..

**A/eq/Richmond/1/2007**  **THPSSSAGLKDDLLENLQAYQKRMGVQMQRFK**

**A/eq/Perthshire/3/2009**  **................................**

**A/eq/Yorkshire/3/2009**  **................................**

**A/eq/Shropshire/2010**  **................................**

**A/eq/Neuville-Pres-Sees/1/2011** **................................**

**A/eq/Devon/1/2011**  **................................**

**A/eq/Northamptonshire/1/2013**  **................................**

**A/eq/Gironde/1/2014**  **................................**

**A/eq/Buckinghamshire/1/2014 ................................**

**A/eq/Kent/1/2015 ................................**

**A/eq/East Sussex/1/2015**  **................................**

**A/eq/East Renfrewshire/2/2011 ................................**

**A/eq/Sweden/VIR160172/2011**  **................................**

**A/eq/Cambremer/1/2012**  **................................**

**A/eq/Ankara/1/2013**  **................................**

**A/eq/Saone-et-Loire/1/2015**  **................................**

**3.10 M2**

10 20 30 40 50 60 70 80 90

....|....|....|....|....|....|....|....|....|....|....|....|....|....|....|....|....|....|....|..

**A/eq/Richmond/1/2007**  **MSLLTEVETPTRNGWECKCSDSSDPLVIAASIIGILHLILWILDRLFFKFIYRRLKYGLKRGPSTEGVPESMREEYRQEQQNAVSVDDSHFVNIELE**

**A/eq/Perthshire/3/2009**  **....................G............................................................................**

**A/eq/Yorkshire/3/2009**  **....................G.................................I............I.............................**

**A/eq/Shropshire/2010**  **....................G..............................................I.............................**

**A/eq/Neuville-Pres-Sees/1/2011** **....................G..............................................I.............................**

**A/eq/Devon/1/2011**  **....................G..............................................I.............................**

**A/eq/Northamptonshire/1/2013**  **....................G..............................................I.............................**

**A/eq/Gironde/1/2014**  **....................G..............................................I.............................**

**A/eq/Buckinghamshire/1/2014 ....................G..............................................I.............................**

**A/eq/Kent/1/2015 ....................G..............................................I.............................**

**A/eq/East Sussex/1/2015**  **....................G..............................................I.............................**

**A/eq/East Renfrewshire/2/2011 ....................G................................H...........................................**

**A/eq/Sweden/VIR160172/2011** **..........................................................M.........................D...G......R.**

**A/eq/Cambremer/1/2012**  **----................G................................H...............K................-----------**

**A/eq/Ankara/1/2013**  **....................G..............................................I.............................**

**A/eq/Saone-et-Loire/1/2015**  **----................G................................H...............K.......................L...**

**3.11 NS1**

10 20 30 40 50 60 70 80 90 100 110

....|....|....|....|....|....|....|....|....|....|....|....|....|....|....|....|....|....|....|....|....|....|

**A/eq/Richmond/1/2007**  **MDSNTVSSFQVDCFLWHVRKRFADQELGDAPFLDRLRRDQKSLRGRGITLGLDIETATHAGKQIVEQILEKESDEALKMTIASIPTSRYLTDMTLDEMSRDWFMLMPKQK**

**A/eq/Perthshire/3/2009**  **...............................................S.................K.................V..........................**

**A/eq/Yorkshire/3/2009**  **..............................................................................................................**

**A/eq/Shropshire/2010**  **..............................................................................................................**

**A/eq/Neuville-Pres-Sees/1/2011** **..............................................................................................................**

**A/eq/Devon/1/2011**  **..............................................................................................................**

**A/eq/Northamptonshire/1/2013**  **......L........................................................................................E..............**

**A/eq/Gironde/1/2014**  **......L........................................................................................E..............**

**A/eq/Buckinghamshire/1/2014 ......L........................................................................................E..............**

**A/eq/Kent/1/2015 ......L..........................................................K.............................E..............**

**A/eq/East Sussex/1/2015**  **......L.......................................................................K................E..............**

**A/eq/East Renfrewshire/2/2011 ..............................................................................................................**

**A/eq/Sweden/VIR160172/2011**  **...............................................S.................K.................V..........................**

**A/eq/Cambremer/1/2012**  **.........................................................................................S....................**

**A/eq/Ankara/1/2013**  **................N........G.....................................................I..............................**

**A/eq/Saone-et-Loire/1/2015**  **.....................................................................................I........................**

120 130 140 150 160 170 180 190 200 210

....|....|....|....|....|....|....|....|....|....|....|....|....|....|....|....|....|....|....|....|....|....

**A/eq/Richmond/1/2007**  **VTGSLCIRMDQAIMDKNIILKANFSVIFERLETLILLRAFTEEGAVVGEISPLPSLPGHTNEDVKNAIGVLIGGLKWNDNTVRISETLQRFAWRSSYENGRPSFPSKQK**

**A/eq/Perthshire/3/2009**  **................................................................................................H............**

**A/eq/Yorkshire/3/2009**  **................................................................................................H............**

**A/eq/Shropshire/2010**  **................................................................................................H............**

**A/eq/Neuville-Pres-Sees/1/2011** **.....................................................................I..........................H............**

**A/eq/Devon/1/2011**  **.....................................................................I..........................H..........--**

**A/eq/Northamptonshire/1/2013**  **.....................................................................I..........................H............**

**A/eq/Gironde/1/2014**  **.....................................................................I..........................H............**

**A/eq/Buckinghamshire/1/2014 .....................................................................I..........................H............**

**A/eq/Kent/1/2015 .....................................................................I.....................T....H............**

**A/eq/East Sussex/1/2015**  **.......................................................F.............I..E.......................H............**

**A/eq/East Renfrewshire/2/2011 ................................................................................................H............**

**A/eq/Sweden/VIR160172/2011**  **................................................................................................N.I..........**

**A/eq/Cambremer/1/2012**  **......................................................................................A.........H............**

**A/eq/Ankara/1/2013**  **A...............................................................................................H............**

**A/eq/Saone-et-Loire/1/2015**  **......................................................................................A.........H............**

**3.12 NEP**

10 20 30 40 50 60 70 80 90 100 110

....|....|....|....|....|....|....|....|....|....|....|....|....|....|....|....|....|....|....|....|....|....|

**A/eq/Richmond/1/2007**  **MDSNTVSSFQDILMRMSKMQLGSSSEDLNGMIIRLESLKLYRDSLGEAVMRMGDLHSLQSRNEKWREQLSQKFEEIRWLIEEVRHRLKNTENSFEQITFMQALQLLLEVE**

**A/eq/Perthshire/3/2009**  **..............................................................................................................**

**A/eq/Yorkshire/3/2009**  **..............................................................................................................**

**A/eq/Shropshire/2010**  **..............................................................................................................**

**A/eq/Neuville-Pres-Sees/1/2011** **..............................................................................................................**

**A/eq/Devon/1/2011**  **..............................................................................................................**

**A/eq/Northamptonshire/1/2013**  **......L.......................................................................................................**

**A/eq/Gironde/1/2014**  **......L......T................................................................................................**

**A/eq/Buckinghamshire/1/2014 ......L......T................................................................................................**

**A/eq/Kent/1/2015 ......L......T................................................................................................**

**A/eq/East Sussex/1/2015**  **......L......T...........K.......................................K............................................**

**A/eq/East Renfrewshire/2/2011 ..............................................................................................................**

**A/eq/Sweden/VIR160172/2011**  **...................................................L..........................................................**

**A/eq/Cambremer/1/2012**  **..............................................................................................................**

**A/eq/Ankara/1/2013**  **.....................................................................................G........................**

**A/eq/Saone-et-Loire/1/2015**  **..............................................................................................................**

120

....|....|.

**A/eq/Richmond/1/2007**  **QEIRTFSFQLI**

**A/eq/Perthshire/3/2009**  **...........**

**A/eq/Yorkshire/3/2009**  **...........**

**A/eq/Shropshire/2010**  **...........**

**A/eq/Neuville-Pres-Sees/1/2011** **...........**

**A/eq/Devon/1/2011**  **...........**

**A/eq/Northamptonshire/1/2013**  **...........**

**A/eq/Gironde/1/2014**  **...........**

**A/eq/Buckinghamshire/1/2014 ...........**

**A/eq/Kent/1/2015 ...........**

**A/eq/East Sussex/1/2015**  **...........**

**A/eq/East Renfrewshire/2/2011 ...........**

**A/eq/Sweden/VIR160172/2011**  **..........M**

**A/eq/Cambremer/1/2012**  **...........**

**A/eq/Ankara/1/2013**  **...........**

**A/eq/Saone-et-Loire/1/2015**  **...........**
