# Supplementary material for: Whole‐genome sequencing and antigenic analysis of the first equine influenza virus identified in Turkey
Source: Influenza Other Respir Viruses. 2018 Feb 8;12(3):374–82. doi: 10.1111/irv.12485 (PMC5907808; doi:10.1111/irv.12485)
Supplement: Supplementary file 3 [file IRV-12-374-s003.docx]

Supplementary Table 1: Accession codes for EIV HA1 and NA sequences included in phylogenetic analysis figures 1 and 2, respectively. GISAID accession numbers are highlighted in bold.

| **Year** | **Country** | **Isolate Name** | **HA1**  **accession** | **NA**  **accession** |
| --- | --- | --- | --- | --- |
| 1963 | USA | A/equine/Miami/1963 | M29257.1 | CY028838.1 |
| 1979 | France | A/equine/Fontainbleu/1/1979 | CY032405.1 | CY032407.1 |
| 1979 | United Kingdom | A/equine/NewMarket/1/1979 | KJ643908.1 | CY096893.1 |
| 1983 | USA | A/equine/New York/VR-297/1983 | / | CY028918.1 |
| 1989 | Germany | A/equine/Berlin/1/1989 | CY032413.1 | / |
| 1989 | Ireland | A/equine/Kildare/1/1989 | JN222941.1 | / |
| 1991 | USA | A/equine/Kentucky/1/1991 | / | CY030175.1 |
| 1992 | Hong Kong | A/equine/Hong Kong/1/1992 | L27597.1 | / |
| 1992 | USA | A/equine/Kentucky/1/1992 | / | CY030151.1 |
| 1992 | Ireland | A/equine/Kildare/1/1992 | JN084402.1 | / |
| 1993 | United Kingdom | A/equine/Newmarket/1/1993 | X85088.2 | FJ375222.1 |
| 1993 | United Kingdom | A/equine/Newmarket/2/1993 | X85089.2 | FJ375223.1 |
| 1994 | Argentina | A/equine/Argentina/1/1994 | JN084406.1 | / |
| 1994 | USA | A/equine/Kentucky/8/1994 | / | CY030183.1 |
| 1998 | USA | A/equine/Kentucky/1/1998 | AF197241.1 | / |
| 2003 | United Kingdom | A/equine/Newmarket/5/2003 | FJ375213.1 | / |
| 2003 | USA | A/equine/Ohio/1/2003 | DQ124192.1 | / |
| 2003 | USA | A/equine/South Africa/4/2003 | GU447312.1 | / |
| 2005 | United Kingdom | A/equine/Aboyne/1/2005 | EF541442.1 | KF049177.1 |
| 2006 | United Kingdom | A/equine/Lincolnshire/2006 | / | KF049176.1 |
| 2007 | Japan | A/equine/Ibaraki/1/2007 | AB360549.2 | / |
| 2007 | United Kingdom | A/equine/Lincolnshire/1/2007 | FJ195398.2 | KF559342.1 |
| 2007 | Ireland | A/equine/Meath/1/2007 | JN222935.1 | / |
| 2007 | United Kingdom | A/equine/Richmond/1/2007 | FJ195395.3 | KF559336.1 |
| 2007 | Spain | A/equine/Spain/1/2007 | CY075851.1 | CY075852.2 |
| 2007 | Switzerland | A/equine/Switzerland/P112/2007 | FJ195408.1 | **EPI584118** |
| 2008 | Ireland | A/equine/Down/1/2008 | JN222937.1 | / |
| 2008 | Poland | A/equine/Pulawy/1/2008 | KT429521.1 | / |
| 2009 | Ireland | A/equine/Carlow/1/2009 | JN222939.1 | / |
| 2009 | Ireland | A/equine/Donegal/1/2009 | JN222938.1 | / |
| 2009 | United Kingdom | A/equine/Dorset/1/2009 | CY054287.1 | KF049196.1 |
| 2009 | United Kingdom | A/equine/Lanarkshire/2009 | / | **EPI584197** |
| 2009 | United Kingdom | A/equine/Perthshire/3/2009 | **EPI584211** | **EPI584213** |
| 2009 | Spain | A/equine/Spain/1/2009 | CY075849.1 | / |
| 2009 | United Kingdom | A/equine/Yorkshire/3/2009 | **EPI584219** | **EPI584221** |
| 2010 | USA | A/equine/California/1/2010 | / | **EPI584272** |
| 2010 | Germany | A/equine/Hamburg/2010 | CY092814.1 | / |
| 2010 | Ireland | A/equine/Kildare/2/2010 | KC871537.1 | / |
| 2010 | Ireland | A/equine/Limerick/1/2010 | JN222940.1 | / |
| 2010 | France | A/equine/Marcy-l'Etoile/1/2010 | JX091786.1 | / |
| 2010 | United Kingdom | A/equine/Shropshire/2010 | **EPI584227** | **EPI584229** |
| 2011 | Ireland | A/equine/Carlow/2011 | KC871546.1 | / |
| 2011 | United Kingdom | A/equine/Devon/1/2011 | KF026389.1 | KF049194.1 |
| 2011 | United Kingdom | A/equine/East Renfrewshire/2/2011 | **EPI584236** | **EPI584238** |
| 2011 | Germany | A/equine/Ittlingen/1/2011 | CY107012.1 | / |
| 2011 | USA | A/equine/Kentucky/1/2011 | / | **EPI670002** |
| 2011 | Ireland | A/equine/Kilkenny/2011 | KC871545.1 | / |
| 2011 | Mongolia | A/equine/Mongolia/3/2011 | JX549062.1 | / |
| 2011 | France | A/equine/Neuville-Pres-Sees/1/2011 | KY241316.1 | KY241347.1 |
| 2011 | Sweden | A/equine/Sweden/SVA111128SZ0073/  VIR160172/2011 | **EPI594524** | / |
| 2011 | Algeria | A/equine/Tiaret/1/2011 | KF317697.1 | / |
| 2012 | France | A/equine/Cambremer/1/2012 | KY241317.1 | KY241351.1 |
| 2012 | United Kingdom | A/equine/County Durham/2/2012 | KF026396.1 | KF049192.1 |
| 2012 | Dubai | A/equine/Dubai/1/2012 | KF026411.1 | KF049173.1 |
| 2012 | USA | A/equine/Kentucky/2/2012 | KF026408.1 | / |
| 2012 | Ireland | A/equine/Kildare/2/2012 | **EPI526643** | / |
| 2012 | Germany | A/equine/Lichtenfeld/1/2012 | JX499136.1 | KF049191.1 |
| 2012 | Germany | A/equine/Rastatt/1/2012 | KC584975.1 | / |
| 2012 | Brazil | A/equine/Rio Grande do Sul/1/2012 | / | **EPI584297** |
| 2012 | Kazakhstan | A/equine/South Kazakhstan/236/2012 | KF712451.1 | / |
| 2012 | United Kingdom | A/equine/Worcestershire/1/2012 | / | KF049174.1 |
| 2013 | China | A/equine/Heilongjiang/SS1/2013 | KC986390.2 | / |
| 2013 | United Kingdom | A/equine/Lanarkshire/1/2013 | / | **EPI493613** |
| 2013 | United Kingdom | A/equine/Northamptonshire/1/2013 | **EPI492817** | **EPI493625** |
| 2013 | United Kingdom | A/equine/Northamptonshire/5/2013 | / | **EPI838917** |
| 2013 | United Kingdom | A/equine/Shropshire/7/2013 | / | **EPI493624** |
| 2014 | United Kingdom | A/equine/Buckinghamshire/1/2014 | **EPI651398** | **EPI651400** |
| 2014 | France | A/equine/Gironde/1/2014 | KY241319.1 | KY241352.1 |
| 2014 | Germany | A/equine/North Rhine Westphalia/1/2014 | KJ538149.1 | / |
| 2014 | Italy | A/equine/Rome/1/2014 | KR534268.1 | / |
| 2015 | United Kingdom | A/equine/East Sussex/1/2015 | **EPI873584** | **EPI873585** |
| 2015 | United Kingdom | A/equine/Kent/1/2015 | **EPI873593** | **EPI873592** |
| 2015 | France | A/equine/Saone-et-Loire/1/2015 | KY241320.1 | KY241353.1 |

Supplementary Table 2: Accession codes for sequences used in Table 2 and supplementary alignments. GISAID accession numbers are highlighted in bold.

|  |  |  | **Segment and Segment Accession** | | | | | | | |
| --- | --- | --- | --- | --- | --- | --- | --- | --- | --- | --- |
| **Source** | **Country** | **Isolate Name** | **PB2** | **PB1** | **PA** | **HA** | **NP** | **NA** | **MP** | **NS** |
| NCBI | UK | Richmond/1/2007 | KF559332.1 | KF559333.1 | KF559334.1 | FJ195395.3 | KF559335.1 | KF559336.1 | KF559337.1 | FJ195429.1 |
| NCBI | France | Neuville-Pres-Sees/1/2011 | KY241368.1 | KY241374.1 | KY241380.1 | KY241316.1 | KY241322.1 | KY241347.1 | KY241359.1 | KY241362.1 |
| NCBI | France | Cambremer/1/2012 | KY241369.1 | KY241375.1 | KY241381.1 | KY241317.1 | KY241323.1 | KY241351.1 | KY241356.1 | KY241363.1 |
| NCBI | France | Gironde/1/2014 | KY241370.1 | KY241376.1 | KY241382.1 | KY241319.1 | KY241324.1 | KY241352.1 | KY241357.1 | KY241364.1 |
| NCBI | France | Saone-et-Loire/1/2015 | KY241371.1 | KY241377.1 | KY241383.1 | KY241320.1 | KY241325.1 | KY241353.1 | KY241358.1 | KY241365.1 |
| GISAID | UK | Perthshire/3/2009 | **EPI584208** | **EPI584209** | **EPI584210** | **EPI584211** | **EPI584212** | **EPI584213** | **EPI584214** | **EPI584215** |
| GISAID | UK | Yorkshire/3/2009 | **EPI584216** | **EPI584217** | **EPI584218** | **EPI584219** | **EPI584220** | **EPI584221** | **EPI584222** | **EPI584223** |
| GISAID | UK | Shropshire/2010 | **EPI584224** | **EPI584225** | **EPI584226** | **EPI584227** | **EPI584228** | **EPI584229** | **EPI584230** | **EPI584231** |
| GISAID | UK | Devon/1/2011 | **EPI584241** | **EPI584242** | **EPI584243** | **EPI584244** | **EPI584245** | **EPI584246** | **EPI584247** | **EPI584248** |
| GISAID | UK | East Renfrewshire/2/2011 | **EPI584233** | **EPI584234** | **EPI584235** | **EPI584227** | **EPI584237** | **EPI584238** | **EPI584239** | **EPI584240** |
| GISAID | Sweden | Sweden/SVA111128SZ0073  /VIR160172/2011 | **EPI594819** | **EPI594796** | **EPI594758** | **EPI594524** | **EPI594745** | **EPI594822** | **EPI594561** | **EPI594744** |
| GISAID | UK | Northamptonshire/1/2013 | **EPI584249** | **EPI584250** | **EPI584251** | **EPI492817** | **EPI584252** | **EPI493625** | **EPI584254** | **EPI584259** |
| GISAID | UK | Buckinghamshire/1/2014 | **EPI651396** | **EPI662160** | **EPI651397** | **EPI651398** | **EPI651399** | **EPI651400** | **EPI651401** | **EPI651402** |
| GISAID | UK | Kent/1/2015 | **EPI873586** | **EPI873587** | **EPI873588** | **EPI873593** | **EPI873590** | **EPI873592** | **EPI873591** | **EPI873589** |
| GISAID | UK | East Sussex/1/2015 | **EPI873607** | **EPI873608** | **EPI873580** | **EPI873584** | **EPI873582** | **EPI873585** | **EPI873583** | **EPI873581** |

Supplementary Table 3: Accession codes for PA-X protein sequences used in alignments. GISAID accession numbers are highlighted in bold.

| **Source** | **Country** | **Isolate Name** | **PA-X** |
| --- | --- | --- | --- |
| NCBI | UK | Richmond/1/2007 | KF559334.1 |
| NCBI | France | Neuville-Pres-Sees/1/2011 | APQ31522.1 |
| NCBI | France | Cambremer/1/2012 | APQ31524.1 |
| NCBI | France | Gironde/1/2014 | APQ31526.1 |
| NCBI | France | Saone-et-Loire/1/2015 | APQ31528.1 |
| GISAID | UK | Perthshire/3/2009 | **EPI507495** |
| GISAID | UK | Yorkshire/3/2009 | **EPI507496** |
| GISAID | UK | Shropshire/2010 | **EPI507499** |
| GISAID | UK | Devon/1/2011 | **EPI507500** |
| GISAID | UK | East Renfrewshire/2/2011 | **EPI507501** |
| GISAID | Sweden | Sweden/SVA111128SZ0073  /VIR160172/2011 | **EPI594758** |
| GISAID | UK | Northamptonshire/1/2013 | **EPI507502** |
| GISAID | UK | Buckinghamshire/1/2014 | **EPI651397** |
| GISAID | UK | Kent/1/2015 | **EPI873588** |
| GISAID | UK | East Sussex/1/2015 | **EPI873580** |
